# Construction of a glycosylation-related prognostic signature for predicting prognosis, tumor microenvironment, and immune response in soft tissue sarcoma

**DOI:** 10.3389/fonc.2025.1636830

**Published:** 2025-09-02

**Authors:** Ningning Yuan, Jinfeng Zhang, Junhui Yuan, Zhou Xie, Yuanjun Gao, Muqaddas Raza, Yutong Gao, Guowei Zuo

**Affiliations:** ^1^ Key Laboratory of Diagnostic Medicine Designated by the Chinese Ministry of Education, Department of Laboratory Medicine, Chongqing Medical University, Chongqing, China; ^2^ Department of Clinical Laboratory, Qingdao Women and Children’s Hospital, Qingdao, Shandong, China; ^3^ Department of Breast &Thyroid Surgery, Qingdao Women and Children’s Hospital, Qingdao, Shandong, China; ^4^ Department of Orthopedics, The First Affiliated Hospital of Chongqing Medical University, Chongqing, China

**Keywords:** soft tissue sarcoma, glycosylation, machine learning, tumor microenvironment, bioinformatics

## Abstract

**Background:**

Altered glycosylation, one of the most common post-translational protein modifications, plays a critical role in the initiation and progression of soft tissue sarcoma (STS). Dysregulated expression of glycosyltransferases leads to abnormal glycosylation patterns, which may offer valuable insights for prognosis and therapeutic response prediction in STS.

**Methods:**

Transcriptional variants and expression profiles of glycosylation-related genes were analyzed using data from The Cancer Genome Atlas (TCGA). Differential gene expression analysis and non-negative matrix factorization (NMF) were performed to identify STS molecular subtypes. A comprehensive machine learning framework integrating 101 algorithms was applied to construct a glycosyltransferase-based prognostic signature. Kaplan–Meier analysis, Cox regression, and receiver operating characteristic (ROC) curves were used to assess the prognostic value of the model. Immune infiltration was evaluated using multiple computational approaches, and functional validation was conducted via *in vitro* experiments.

**Results:**

Two distinct STS subtypes with significant immunological and clinical differences were identified. A 12-gene glycosyltransferase signature was developed, effectively stratifying patients into high-risk and low-risk groups based on the median riskscore. The high-risk group demonstrated significantly poorer survival outcomes. Immune profiling revealed greater immunosuppression in the high-risk group. *In vitro* silencing of STT3A significantly suppressed proliferation and migration of STS cells.

**Conclusions:**

The proposed glycosylation-related gene signature accurately distinguishes between high- and low-risk STS patients and may serve as a reliable prognostic tool. It also provides novel insights into tumor immune microenvironment and potential therapeutic targets for STS.

## 1 Introduction

Soft tissue sarcoma is a rare mesenchymal malignancy, accounting for 1% of all adult cance (1). The rarity and diverse initial clinical and histological presentations of STS make its diagnosis particularly challenging. STS is highly aggressive and exhibits poor responsiveness to systemic therapies, which traditionally rely on anthracycline-based chemotherapy tailored according to factors such as histotype, grading, and tumor location. Surgical intervention has been shown to improve outcomes in patients with localized disease. However, despite optimal local treatment, up to 40% of STS patients develop metastatic disease, which often leads to fatal progression (2). Although survival rates have gradually improved over the past two decades, progress still remains limited, due in large part to the paucity of innovative treatment approaches and the difficulties in carrying out extensive, comprehensive research in such a rare and heterogeneous cancer population (3). To date, immunotherapy, particularly immune checkpoint inhibitors (ICIs), has shown effective in STS (4). However, the complicated tumor microenvironment (TME) of STS reduces clinical response rates to current mainstream pharmacological treatments. The interaction between tumor cells and the surrounding microenvironment is closely associated with tumorigenesis and immune evasion (5). It is crucial to comprehensively explore the hidden information within the TME and integrate it with innovative immunotherapeutic strategies. In order to support personalized treatment and accurate medicine, it is imperative to uncover novel markers to forecast immunotherapy responses and to develop reliable prognostic signatures for individuals with STS.

Glycosylation is the most abundant and diversified type of post-translational modification shared by all eukaryotic cells, involving glycosylation enzymes from many families, including glycosyltransferases and glycosidases (6). Advances in glycoproteomics linked to cancer have given light on the pathways that underpin tumor genesis and dissemination (6). O-glycan truncation, sialylation, fucosylation, and N-glycan branching are the most common alterations in cancer-associated glycosylation (6). These modifications promote a variety of malignant tumor behaviors, which include tumor cell proliferation, invasion, metastasis, angiogenesis, immunological modulation, and the development of treatment resistance (7). Dysregulated glycosylation expression impacts chemotherapy-induced cell apoptosis via multiple pathways, including the PI3K/Akt and p38 MAPK/caspase signaling cascade (8, 9). This highlights the significant potential of glycosylation biomarkers and their expression changes as diagnostic and prognostic biomarkers for soft tissue sarcoma, as well as in the context of therapy resistance.

To our knowledge, few studies have systematically investigated the differential expression of glycosylation-related genes in the tumor microenvironment of STS using integrated bulk RNA sequencing and large-scale transcriptomic datasets. Our study not only fills this gap but also establishes a glycosylation-based prognostic model with potential clinical utility. In this study, we initially screened 12 glycosylation related genes that were differently expressed based on the genomic information of STS samples and developed a prognostic risk model accordingly. It was successful to divide STS cases into low-risk and high-risk groups based on the median riskscore. These two groups exhibited notable differences in overall survival (OS), gene expression, immune infiltration, immune checkpoint inhibitor response, and drug sensitivity. Furthermore, *in vitro* assays demonstrated that the chosen glycosylation related gene have been linked to the malignant characteristics of STS. Our findings indicate that the glycosylation-based model can serve as a predictive tool for the prognosis and immune status of individuals with STS. This study provides an additional screening method to guide prognosis assessment and immunotherapy for STS.

## 2 Materials and methods

### 2.1 Data acquisition and processing

**Figure 1:**
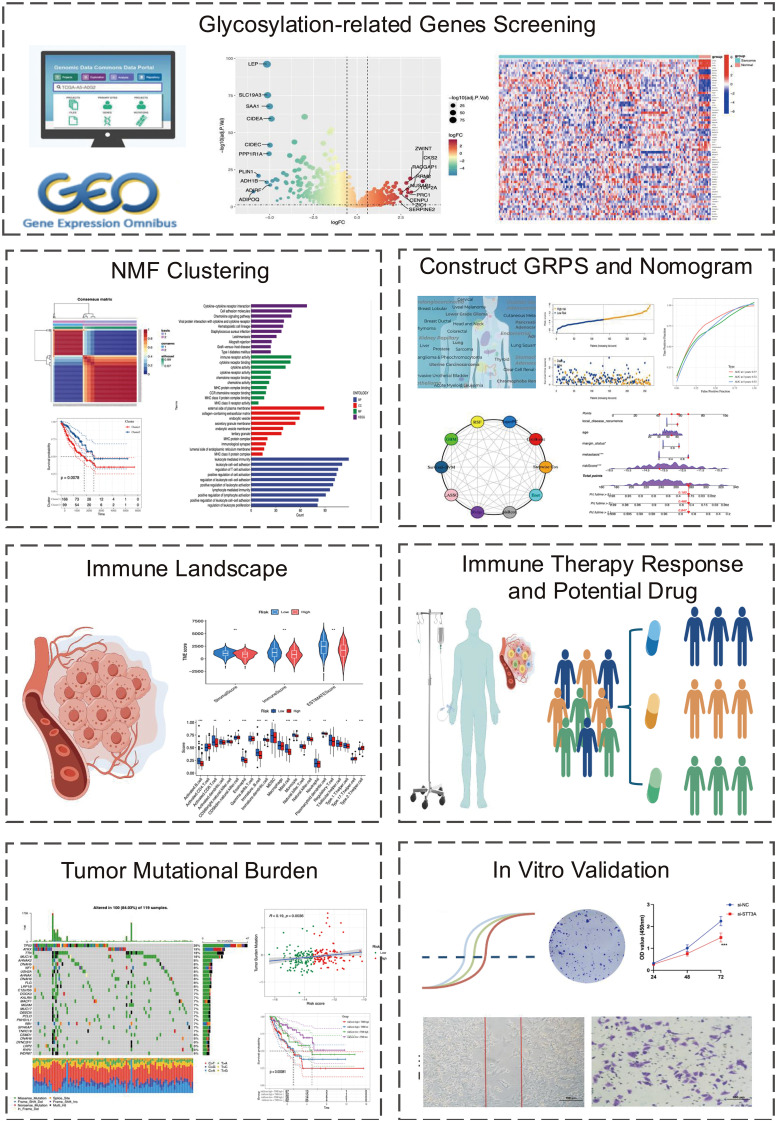
The workflow of this study.

This research utilized data from two separate cohorts to analyze the transcriptomic characteristics of STS. The training set comprised 265 STS cases sourced from The Cancer Genome Atlas (TCGA) database (https://portal.gdc.cancer.gov/) were used as the training set. This dataset encompasses comprehensive clinical data, such as gender, age, metastatic status, and the locations of both primary and specific tumors. To ensure independent validation, the GSE17674 dataset from the Gene Expression Omnibus (GEO) database (https://www.ncbi.nlm.nih.gov/geo/) was used, containing 32 STS cases along with corresponding clinical attributes, such as gender, age, metastasis status, and tumor location. Additionally, differential analysis was performed using the GSE21122 dataset from GEO, which includes 9 normal tissue samples and 149 STS tissue samples. This dataset aids in investigating the gene expression changes between normal tissues and STS tissues. Based on existing literature, 240 glycosylation -related genes (GRGs) were selected. The list of glycosylation-related genes was compiled based on Reference (10), and is provided in **Supplementary Table 1**. Analyzing these datasets identified differentially expressed GRGs in STS tissues versus normal tissues, shedding light on their potential roles in the pathogenesis of STS. The workflow of this study was shown in **Figure 1**.

### 2.2 Differentially expressed GRGs in STS

The GSE21122 dataset was subjected to differential expression analysis between normal tissue samples and STS tissue samples using the R programming environment’s limma package. A significance threshold with an adjusted *P*-value < 0.05 was applied to identify differentially expressed genes (DEGs) between STS and normal tissues. To identify GRGs associated with STS, we analyzed the intersection of a comprehensive set of 240 GRGs and the DEGs. The resulting genes were termed STS-related differentially expressed GRGs.

### 2.3 Identification of differentially expressed genes and analysis using non-negative matrix factorization algorithm

The molecular subtypes of STS patients in the training cohort were examined utilizing the “NMF” R package, which was based on the differentially expressed GRGs. The ideal number of clusters (k) was chosen by assessing cophenetic, dispersion, and silhouette metrics. Ultimately, k = 2 was selected, resulting in the classification of patients into two distinct molecular clusters. Survival curves for these two clusters were calculated using the “survival” R package.

### 2.4 Enrichment analysis of GO and KEGG

To further investigate the biological functions associated with different molecular subtypes, we conducted GO and KEGG enrichment analysis on the DEGs across the NMF clusters using the “clusterProfiler” R package. Through this analysis, we identified key biological processes, molecular functions, and signaling pathways associated with specific molecular subtypes, providing important insights for subsequent research into the underlying biological mechanisms.

### 2.5 Gene set variation analysis

We performed GSVA on the samples in the training cohort utilizing the “GSVA” R package. GSVA is an unsupervised approach for assessing the variation and activity of gene sets across different samples. We selected predefined HALLMARK gene sets from the GSEA database and scored each sample based on these gene sets. The GSVA score reflects the activity level of a gene set in each sample, with higher scores indicating greater activity of the gene set in that sample.

### 2.6 Construction of a glycosylation -related prognostic signature using integrated machine learning algorithms

In this study, we utilized the “survival” R package to conduct univariate Cox regression analysis on GRGs within the training cohort, identifying differentially expressed GRGs significantly associated with STS prognosis (*P* < 0.01). Subsequently, we developed a Glycosylation -related prognostic signature (GRPS) using an integrated machine learning algorithm approach, applying a combination of 101 machine learning algorithms with 10-fold cross-validation. These algorithms included Lasso, StepCox, Elastic Net (Enet), Ridge, Random Survival Forest (RSF), Partial Least Squares Cox Regression (plsRcox), Cox Boost, Gradient Boosting Machine (GBM), Supervised Principal Component Analysis (SuperPC), and Survival Support Vector Machine (Survival-SVM).

The primary goal was to identify the most valuable prognostic and immunotherapy-related features characterized by the highest concordance index (C-index). The model with the highest C-index was designated as the best GRGs-related feature. The selected model, Lasso + plsRcox, was validated in an independent cohort (GSE17674). We constructed a multivariate Cox regression using GRGs to enhance the interpretability of the prognostic model. The regression coefficients derived from this model were subsequently used to calculate individual riskscore, enabling quantitative stratification of STS patients based on GRGs expression levels.

The risk score for each sample was calculated using the prognostic model formula:


$$\begin{array}{c} Riskscore\;=\;0.109127021\;*XYLT2\;+0.214403\;*STT3A\\ +\;0.103451859\;*PIGC+\;0.044297031\;*B3GAT3\\ -\;0.130121953\;*MFNG+\;0.001495212\;*DDOST\\ +\;0.17580389\;*RPN2\;+0.092825066\;*B4GALT2\\ +\;0.210769724\;*GLT8D1\;+0.246300585\;*EIF2B3\\ +\;0.192022891\;*ALG6\;+0.105703781\;*B3GNT4 \end{array}$$

A higher riskscore is associated with an increased likelihood of death, allowing for the prediction of individual patient survival probabilities.

### 2.7 Validation of glycosylation -related prognostic signature

Regarding the training and validation cohorts, we utilized R to generate various visualization, including risk curves and survival status plots. Survival curves for both cohorts were constructed utilizing the “survival” and “survminer” packages to explore potential survival differences based on cohort type. We employed the “timeROC” package to create ROC curves, illustrating the 1-year, 3-year, and 5-year survival rates of STS patients in both cohorts.

### 2.8 Establishment and validation of the prognostic nomogram

The nomogram is a graphical tool that converts difficult mathematical formulas into an intuitive picture, facilitating multivariable Cox regression analysis for predicting OS in STS patients. Widely used in cancer prognosis, it enables clinicians to estimate survival probability based on numerous clinical factors. This study seeks to create a nomogram for predicting survival outcomes in STS patients by combining major clinical parameters such as gender, main tumor site, metastatic status, specific tumor location, and riskscore. Cox regression analyses, both univariate and multivariate were employed to assess these characteristics and develop a reliable prognostic model.

The “regplot” R tool was utilized to generate an interactive nomogram for predicting the overall survival rates at 1-, 3-, and 5 years. Additionally, calibration plots and ROC curves were used to assess the nomogram’s predictive accuracy. This analysis provides clinicians with valuable insights for more accurate and reliable cancer prognosis prediction.

### 2.9 Single-sample gene set enrichment analysis

To explore the immune cell fraction and functional properties in STS patients, ssGSEA was conducted based on transcriptomic data. Enrichment scores for diverse immune cell types and functions were calculated using the R packages limma, GSVA, and GSEABase. Subsequently, we compared immune cell function between high-risk and low-risk groups in the training cohort to identify differences.

### 2.10 Immune landscape analysis

Studying the tumor microenvironment is crucial for understanding the molecular factors that govern cancer growth and treatment response (11, 12). This study aims to use transcriptome data to assess the tumor microenvironment in STS patients. The study used R software’s estimate tools to calculate STS patients’ stromal score, immunological score, and total score. The stromal score quantifies the relative proportion of stromal cells in the tumor microenvironment, whereas the immune score assesses the degree of immune cell infiltration. Our study investigates the significant differences in stromal score, immune score, and tumor purity between the high-risk and low-risk groups in the training cohort.

### 2.11 Differential analysis of immune checkpoints

To examine the changes in immune checkpoint-related genes within the risk prediction model of the training cohort, statistical software R was used along with the limma, reshape2, ggplot2, and ggpubr packages. The primary goal of this study was to identify the differential expression patterns of immune checkpoint-related genes between the high-risk and low-risk groups. By providing insights into the potential role of immune checkpoint-related genes, the results of this analysis could contribute to the development of prognostic biomarkers and therapeutic strategies for high-risk patients in the training cohort.

### 2.12 Immune function analysis

We used immune function analysis to investigate immune function differences in the training cohort’s various risk categories. This analysis allows us to acquire a better knowledge of the possible involvement of immune function in the prognosis of various risk groups, which wound provide vital hints for the development of immunotherapy treatments and prognostic biomarkers for high-risk patients.

### 2.13 Drug sensitivity analysis

In this study, drug sensitivity related to the risk prediction model was assessed in the training cohort. The R package “oncopredict” and the STS expression data were used for drug sensitivity analysis. GDSC2 was selected as the training dataset to predict the half-maximal inhibitory concentration (IC_50_) of each drug in the high-risk and low-risk groups, and differences between them were compared. Ultimately, drugs with statistically significant IC_50_ values were identified, providing guidance for clinical drug treatment of STS.

### 2.14 Tumor mutation burden evaluation

To examine somatic mutation differences between high-risk and low-risk groups, we analyzed somatic mutations in TCGA using the R package “maftools”. The tumor mutation burden (TMB) of both groups of patients was then estimated. Additionally, waterfall plots were employed to identify the 30 most frequently mutated genes in both high-risk and low-risk categories.

### 2.15 Immunotherapy response

Given the increasing significance of immunotherapy, this study includes the IMvigor210 cohort, comprising patients with advanced urothelial carcinoma treated with anti-PD-L1 antibody atezolizumab. The IMvigor210 cohort can be accessed through the following link: http://research-pub.gene.com/IMvigor210CoreBiologies. DESeq2 was used to normalize the data, followed by TPM annotation with the limma package. Kaplan-Meier survival curves for the immunotherapy cohort were developed using the survival package, with the objective of evaluating the predictive capacity of the riskscore in determining immunotherapy response.

### 2.16 Functional prediction of risk prognostic model genes

Gene functions and interacting proteins for the 12 risk prognostic model genes were predicted using the GeneMania website (https://genemania.org/), a resource offering detailed and reliable predictions of gene functions and interactions.

### 2.17 Cell culture

In this experiment, human rhabdomyosarcoma cell line A673 and human liposarcoma cell line SW872 were purchased from Pricella Biotechnology (China), while Human Bone Marrow-derived MSCs (PCS-500-012) were purchased from ATCC (United States) and used as normal controls for comparison with STS cell lines in gene expression. They were cultured in DMEM (Gibco, USA) with 10% FBS (Gibco, USA) and 1% streptomycin/penicillin (Pricella Biotechnology, China). Cells were cultured at 37°C with 5% CO2. To knock down STT3A, soft tissue sarcoma cell lines were cultured in 6-well plates and transfected with the small interfering RNA (siRNA) from Sangon Biothch (China), using Lipofectamine 3000 (Thermo Fisher Scientific, USA) according to the manufacturer’s instructions for 24 h. The target sequences of siRNA are provided in **Supplementary Table 2**.

### 2.18 RNA extraction and quantitative PCR

RNA was extracted from the cells using TRIzol reagent (Thermo Fisher Scientific, USA) and subsequently reverse-transcribed into single-stranded cDNA with the Evo M-MLV RT Mix Tracking Kit (Accurate Biotechnology, China). qPCR was conducted 10 µL reaction volume according to the manufacturer’s protocol for the SYBR Green Premix Pro Taq HS qPCR Kit (Accurate Biotechnology, China). The primer sequences for the target genes are listed in **Supplementary Table 2**.

### 2.19 Colony formation

A673 and SW872 cells were transfected and seeded into 6-well plates at 1,000 cells per well. After one week of culture, the medium was discarded, and cells were fixed with 4% paraformaldehyde for 15 min, then stained with 0.1% crystal violet. Colonies exceeding 50 cells were quantified using ImageJ software, and the colony formation rate was calculated.

### 2.20 CCK-8 assay

The viability of A673 and SW872 cells was assessed using the CCK-8 assay following the manufacturer’s guidelines (Targetmol, China). Cells were plated at 3,000 per well in 96-well plates, with three replicates for each group. Cells were incubated in the dark for 2 h following the addition of 10 µL of CCK-8 solution to each well. Absorbance was then measured at 450 nm using a microplate reader (Thermo Fisher Scientific, USA).

### 2.21 Transwell assay

The transwell assay evaluated STS cells migration and invasion ability. For invasion, 2.5 × 10^4^ cells in 400 µL serum-free medium placed in Matrigel-coated chambers (Corning, USA), while the lower chamber containing DMEM and 10% FBS. After 24 h incubation at 37°C, cells were fixed, stained with 0.1% crystal violet, and quantified using ImageJ software. The migration assay was performed similarly, without Matrigel, to evaluate cell migration.

### 2.22 Wound healing assay

The wound healing assay assessed the migration capacity of A673 and SW872 cells. When the cell confluence reached 100%, a scratch was gently created in the monolayer using a 200 µL pipette tip. The cells were incubated for 24 h at 37°C in serum-free DMEM medium. The distance between the wound edge and the migrating cells was measured.

### 2.23 Western blotting

STS cells were cultured in 10-cm dishes at 37°C, followed by a 48h transfection period. Post-transfection, cells were trypsinized and harvested, and subsequently lysed using a cell lysis buffer. Protein concentration was determined via the Bicinchoninic Acid method (Beyotime, Institute of Biotechnology, China). After loading the protein samples onto a 10% SDS gel, proteins were separated by 10% SDS-PAGE. Following electrophoresis, the proteins were transferred onto a PVDF membrane. The membrane was incubated overnight at 4°C with primary antibodies against STT3A (Proteintech, China) and β-actin (Proteintech, China), followed by blocking with 5% skim milk. The membrane was washed thrice with 1×TBST and then exposed to an HRP-conjugated secondary antibody (Zsgb-bio, China) for 1 h at room temperature. Protein bands were then visualized and quantified with the Chemidoc MP Imaging System (BioRad, USA).

### 2.24 Statistical analysis

Statistical analysis and visualization were carried out with R software (version 4.3.2) and GraphPad Prism (version 8.2.1). This work used a significance level of *P* < 0.05 to evaluate statistical significance in bioinformatics analysis, which aligns with widely accepted research standards. The experimental data are reported as mean ± SD, and statistical significance was established by one-way ANOVA. *P*-values < 0.05 were considered statistically significant. In addition, each experiment was carried out independently at least three times.

## 3 Results

### 3.1 Differential expression of glycosylation related genes between soft tissue sarcoma and normal tissue

**Figure 2:**
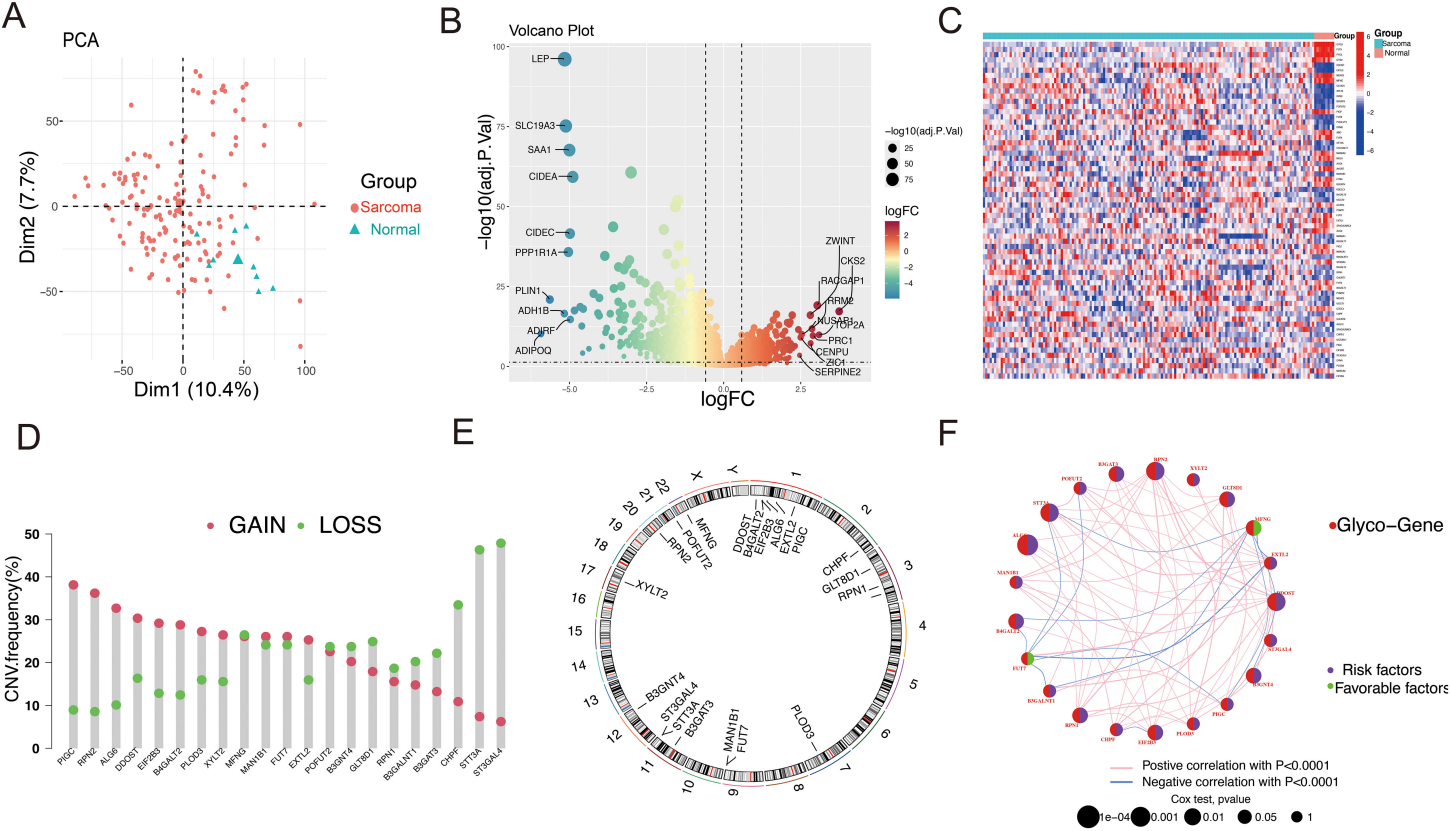
Landscape of the differentially expressed GRGs in STS. **(A)** The STS tissues and normal tissues were clearly distinguished by PCA analysis. **(B)** The volcano plot of DEGs between STS and normal tissues illustrates gene expression differences. In this plot, red dots indicate upregulated genes, while green dots represent downregulated ones. **(C)** The heatmap illustrates the expression patterns of GRGs in sarcoma and normal tissues. Red indicates a high expression level, while blue signifies a low expression level. **(D)** The CNV frequency of GRGs in STS is presented, with bar heights representing the occurrence of variations. The green dot signifies CNV deletions (loss), while the red dot indicated CNV amplification(gain). **(E)** Locations of the 21 differentially expressed GRGs across 23 chromosomes. **(F)** Network of GRGs interactions showing correlations among risk and favorable factors. Red nodes represent glycosylation genes, purple nodes represent risk factors, and green nodes represent beneficial factors. The larger the node, the higher the correlation.

First, Principal component analysis (PCA) analysis was performed on the GSE21122 dataset, revealing a distinct distribution difference between the soft tissue sarcoma and normal tissue groups across the two principal components (**Figure 2A**). Through differential analysis of the GSE21122 dataset, 2,092 DEGs were detected, including 1,100 that were upregulated and 992 that were downregulated. A volcano plot was generated using R software for visualization (**Figure 2B**). Next, an intersection analysis between the DEGs and 240 glycosylation-related genes was carried out, resulting in 65 glycosylation-related differentially expressed genes, which were displayed in a heatmap (**Figure 2C**). Subsequently, univariate cox analysis was conducted to correlate these candidate genes with the prognosis of soft tissue sarcoma patients in the STS dataset of TCGA-SARC, 21 genes were identified.

To explore the genetic alterations of GRGs in soft tissue sarcoma, we analyzed copy number variations (CNVs) (**Figure 2D**). As shown in **Figure 1D**, several glycosylation-related genes (including *PIGC, RPN2, ALG6, DDOST, EIF2B3*, and *B4GALT2*) exhibited increased CNV, while *ST3GAL4, STT3A, CHPF, B3GAT3, B3GALNT1*, and *GLT8D1* showed decreased CNV. The chromosomal locations of these genes were also annotated (**Figure 2E**). The network plot illustrates extensive interactions among glycosylation-related genes (**Figure 2F**). These findings indicate that glycosylation-related genes may be essential for the development and progression of STS.

### 3.2 NMF cluster

**Figure 3:**
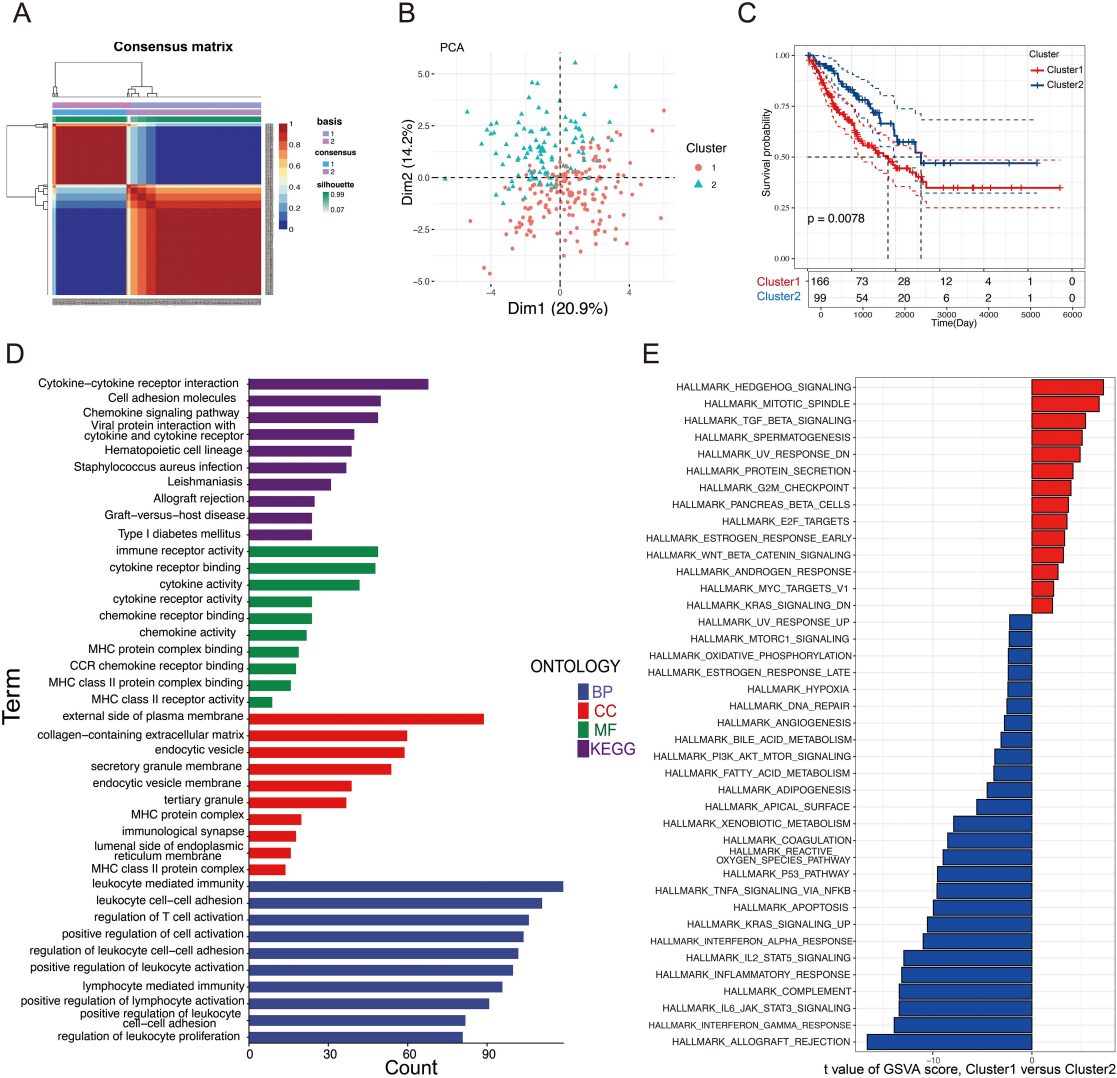
Clusters of differentially expressed GRGs and biological characteristics in STS. **(A)** A consensus matrix that illustrating the outcomes of the non-negative matrix decomposition algorithm, revealing the categorization of patients into two separate subclusters. **(B)** PCA scatter plot demonstrating that the NMF clustering algorithm segmented STS patients into two distinct subclusters. **(C)** KM plots compared the OS of the two subclusters. **(D)** GO and KEGG analysis of the two clusters. **(E)** Analysis of GSVA comparing Cluster 1 and Cluster 2.

Using differentially expressed glycosylation-related genes, NMF clustering was conducted, revealing that the ideal cluster number was 2 (**Supplementary Figures S1A** and **S1B**). Consequently, patients were divided into two separate clusters (**Figure 3A**). PCA demonstrated clear and stable subtype distributions in cluster 1 and cluster 2 (**Figure 3B**). Survival curve analysis indicated that patients in Cluster 1 had notably poorer prognoses than those in Cluster 2 (**Figure 3C**). To uncover potential biological pathways, GO and KEGG pathway enrichment analysis and GSVA were conducted. GO and KEGG pathway analyses revealed possible biological effects between the two clusters. As shown in **Figure 3D**, GO analysis identified enriched GO terms linked to the cytokine-cytokine receptor interaction, immune receptor activity, and the external side of the plasma membrane. In terms of pathway enrichment, the pathways were significantly enriched in lymphocyte-mediated immunity, leucocyte-cell adhesion, and T cell activation regulation. By using GSVA, we observed Cluster 1 was significantly enriched in the Hedgehog signaling pathway, mitotic spindle, and E2F targets, as shown in **Figure 3E**, whereas Cluster 2 was significantly enriched in immune-related pathways, such as allograft rejection, interferon gamma response, and IL-6-JAK-STAT6 signaling. This suggests that Cluster 1 has more pro-tumor activity, whereas Cluster 2 has increased immune activity. The differential activation of these pathways may explain the prognosis differences between these two patient groups.

### 3.3 Development and validation of the prognostic model

Prognostic risk models offer significant potential for precisely forecasting tumor outcomes. To gain deeper insights into the relationship between glycosylation regulators and patient prognosis in STS, univariate Cox regression analysis was conducted, and 21 significant prognostic genes were identified, all of which had *P*-values less than 0.05.

Next, to refine dimensionality further, these genes were filtered using 101 different combinations of 10 machine learning algorithms, ultimately resulting in the development of the prognostic model. These algorithms included Lasso, StepCox, Elastic Net (Enet), Ridge, Random Survival Forest (RSF), Partial Least Squares Cox Regression (plsRcox), Cox Boost, Gradient Boosting Machine (GBM), Supervised Principal Component Analysis (SuperPC), and Survival Support Vector Machine (Survival-SVM). The goal was to construct a prognostic feature related to glycosylation.

**Figure 4:**
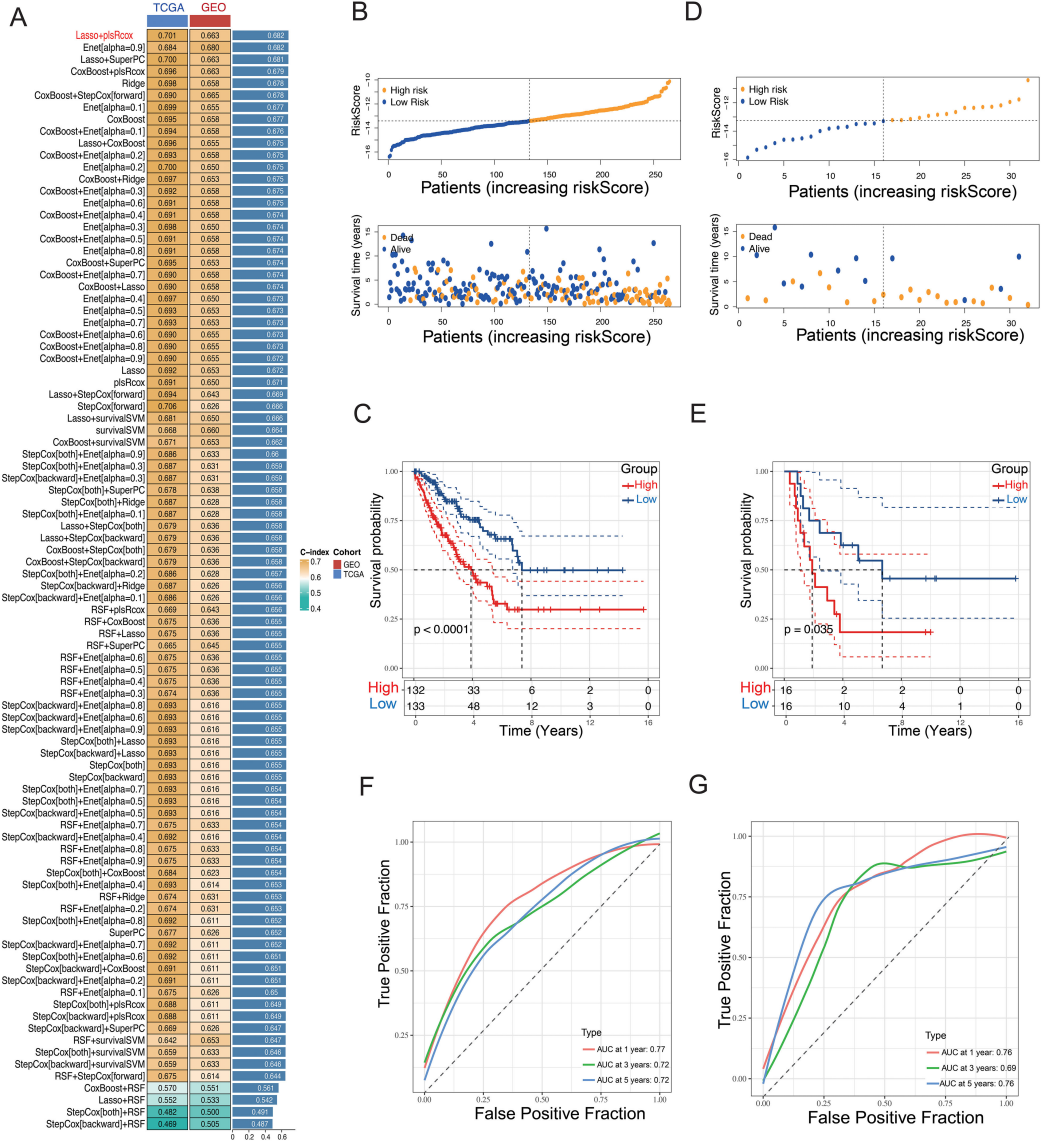
Construction of a GRPS signature. **(A)** A machine-learning integrative approach was employed to formulate and validate a consensus GRPS. **(B, D)** Distribution plots illustrating the correlation between riskscore and OS in the TCGA and GEO cohorts. **(C, E)** Kaplan–Meier analysis of overall survival in the TCGA and GEO cohorts. **(F, G)** ROC analysis of the GRPS for predicting the 1-, 3-, and 5-year OS in the TCGA and GEO cohorts.

The findings demonstrated that the LASSO + plsRcox method exhibited the highest C-index in both cohorts, thus being considered the optimal model for further analysis (**Figure 4A**). Finally, 12 selected glycosylation-related prognostic genes were identified, including *RPN2, ALG6, XYLT2, DDOST, MFNG, PIGC*, EIF2B3, *B3GAT3, B3GNT4*, *B4GALT2, GLT8D1*, and *STT3A*. To enhance model interpretability and compute individualized risk scores, a multivariate Cox regression analysis was performed using the selected genes. Based on the calculated scores, patients were stratified into high- and low-risk groups. The corresponding regression coefficients are presented in **Supplementary Figure S2**. STS patients in both cohorts were classified into high-risk and low-risk groups according to the median riskscore. We observed that, in both the training (TCGA-SARC) and validation cohorts (GSE17674), the high-risk group exhibited a notably greater proportion of deceased patients compared to the low-risk group (**Figures 4B, D**). The riskscore plots and survival status indicated longer survival time for the low-risk group (**Figures 4C, E**). The time-dependent ROC curves highlighted the model’s strong performance in both the training set (5-year AUC = 0.72; 3-year AUC = 0.72; 1-year AUC = 0.77) and the validation set (5-year AUC = 0.76; 3-year AUC = 0.69; 1-year AUC = 0.76) (**Figures 4F, G**). Moreover, Kaplan-Meier and ROC curve analyses (**Supplementary Figure S3**) demonstrated that several signature genes, including *STT3A*, *ALG6*, and *EIF2B3*, were significantly associated with patient survival and showed favorable predictive accuracy.

### 3.4 Construction of the nomogram and evaluation of prognostic signature

**Figure 5:**
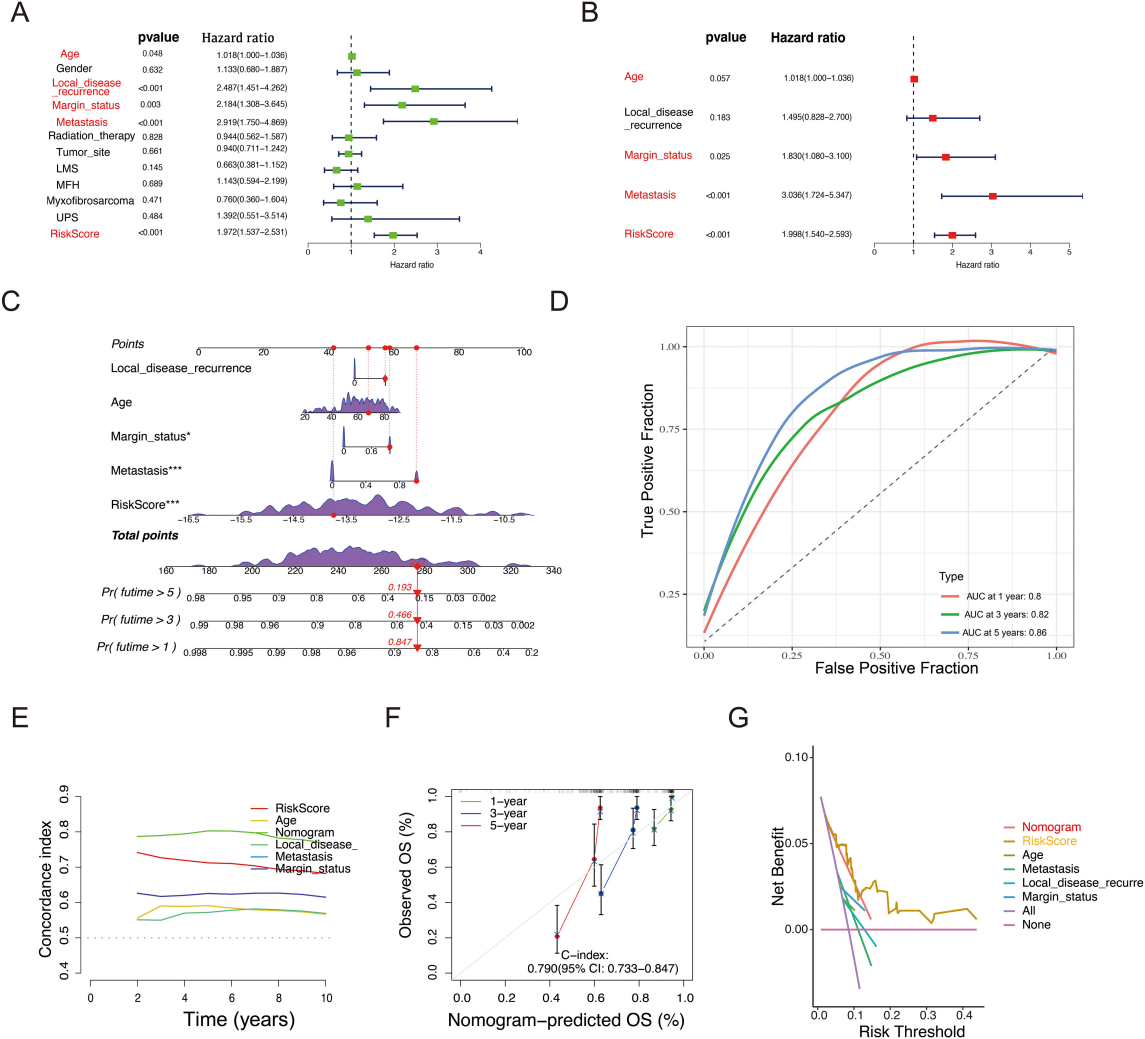
Construction of a nomogram to predict OS for STS patients in the TCGA cohort. **(A, B)** Univariate **(A)** and Multivariate Cox regression **(B)** analysis of the prognostic potential of riskscore alongside other clinicopathological characteristics. **(C)** Development of a nomogram incorporating the riskscore and other clinicopathological factors to predict 1-, 3-, and 5-year OS of STS patients. **(D)** ROC analysis and AUC assessment for the nomogram at 1-, 3-, and 5-years. **(E)** Time-dependent Concordance Index for different variables. **(F)** Calibration curves of the nomogram to validate the alignment between nomogram predictions and actual 1-, 3-, and 5-year survival outcomes for STS. **(G)** DCA of the nomogram.

Clinical characteristics are strongly associated with patient outcomes. To assess whether the riskscore and other clinical factors can independently predict STS prognosis, univariate and multivariate Cox regression analyses were conducted using the training dataset (**Figures 5A, B**). The findings indicated a significant association between metastasis status, riskscore, and prognosis (*P* < 0.05), confirming that the glycosylation-related prognostic signature are robust independent prognostic indicators for STS.

Building on these results, an interactive nomogram was developed to forecast the 1-, 3-, and 5-year overall survival rates for STS patients. To estimate survival probabilities, five lines were drawn upward to assign scores to each factor in the nomogram. The total score, derived by summing these individual scores, was then mapped downward to intersect with the survival axis, allowing for the determination of the predicted survival rates at the specified time points (**Figure 5C**). Additionally, the AUC based on the riskscore and clinical information was higher than 0.8 (**Figure 5D**). **Figure 5E** shows the variation of the Concordance Index (C-index) over time, which helps assess the performance of each variable in predicting survival probability. The nomogram’s C-index further confirmed its strong predictive capability. To assess its accuracy at the 1-, 3-, and 5-year intervals, calibration curves and decision curve analysis (DCA) were employed (**Figures 5F, G**). The calibration curve closely aligned with the ideal curve (gray line in **Figure 5F**), suggesting that the nomogram accurately reflected actual survival outcomes.

### 3.5 Tumor microenvironment analysis

**Figure 6:**
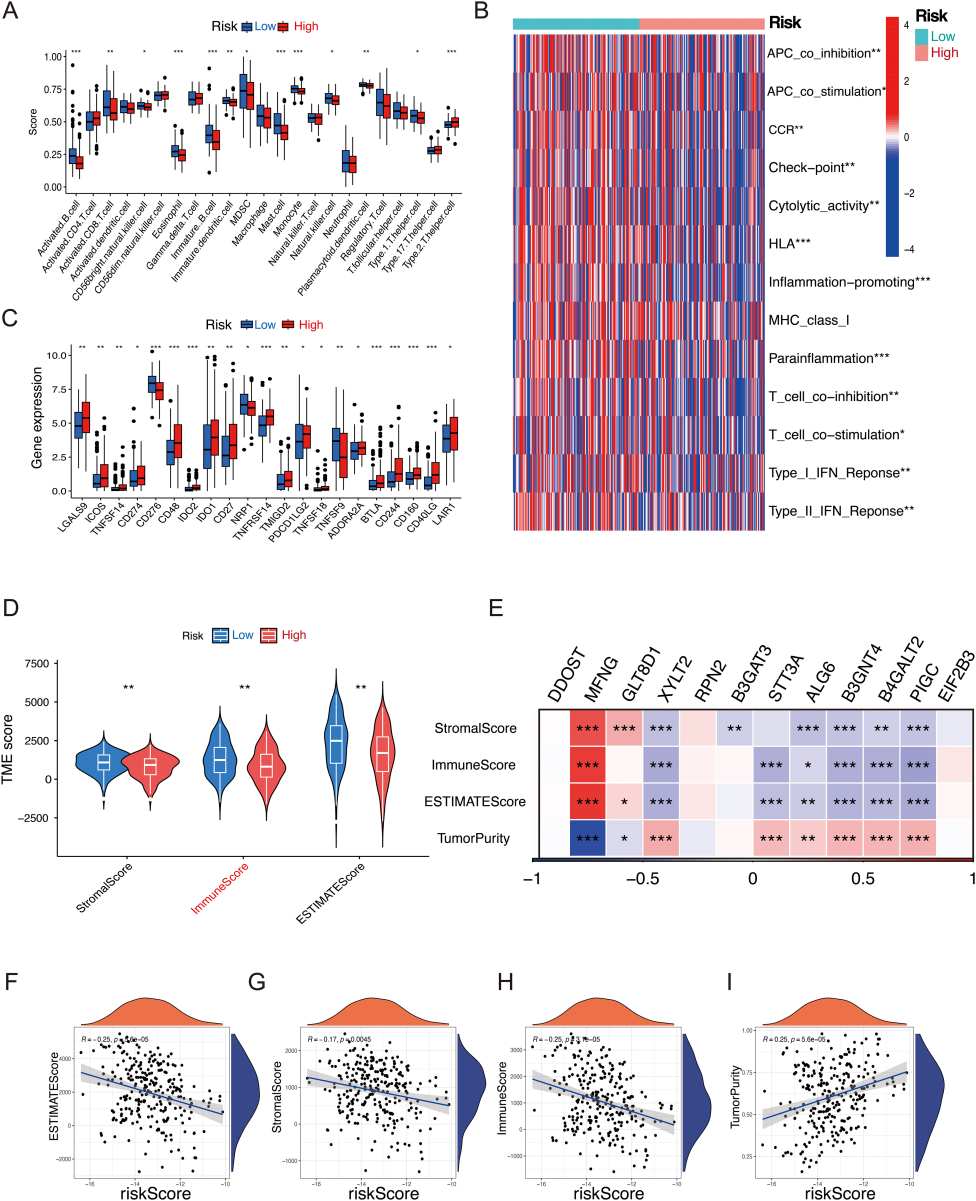
Relationship between the GRPS and the immune landscape of STS patients. **(A)** The ssGSEA algorithm calculated the relative proportion of each immune infiltrating cell in high- and low-risk groups. **(B)** Heatmap of immune function infiltration degree. **(C)** Box plot illustrating the differential expression of immune checkpoints between high- and low-risk groups. **(D)** Violin plots depicting the differences in StromalScore, ImmuneScore, and ESTIMATEScore between high- and low-risk groups. **(E)** Correlation heatmap between twelve GRGs and StromalScore, ImmuneScore, ESTMTEScore and TumorPurity. **(F–I)** Correlation analysis of riskscores and StromalScore, ImmuneScore, ESTMTEScore and TumorPurity. (*P < 0.05, **P < 0.01, ***P < 0.001, ns *P* > 0.05).

As a key mediator of tumor malignancy progression, tumor microenvironment is crucial in processes such as tumor metastasis, immune evasion, and recurrence. To explore significant differences in immune characteristics between the two risk groups, we assessed immune infiltration patterns with ssGESA, and the results revealed that in the low-risk group, activated B cells, activated CD8 T cells, and immature dendritic cells were significantly enriched (**Figure 6A**). Immune function analysis revealed differences in type II IFN Response, APC co-stimulation, CCR, parainflammation, and immune checkpoint activity, indicating that patients in the low-risk group exhibited stronger immune responses (**Figure 6B**). Considering the pivotal role of immune checkpoints in tumor immunotherapy, we checked the expression of immune checkpoints level in both groups and observed the low-risk group exhibited significantly higher expression levels of LGALS9, CD274, CD48, and TNFRSF14 compared to the high-risk group, indicating that the low-risk group could potentially be more responsive to immune checkpoint blockade (**Figure 6C**). Furthermore, the low-risk group exhibited higher immune scores, stromal scores, and ESTIMATE score, indicating that these patients may have a more robust immune response and lower tumor purity within the tumor microenvironment (**Figure 6D**). Additionally, analysis of model genes and ESTIMATE scores revealed that *XYLT2, B3GNT4, B4GALT2, PIGC, and STT3A* were significantly negatively correlated with ESTIMATE score (*P* < 0.01), while MFNG were positively correlated with ESTIMATE score (*P* < 0.01) (**Figure 6E**). The riskscore was negatively correlated with Immune score, Stromal score, and ESTIMATE score, while it showed a positive correlation with tumor purity (**Figures 6F–I**). These results highlight the unique features of the STS tumor microenvironment, providing valuable insights to complement previous studies. In summary, these results imply that patients in the low-risk group show signs of immune activation, whereas those in the high-risk group exhibit immune suppression, suggesting that GRPS features could serve as an effective tool for subtype classification and immune therapy guidance in STS patients.

### 3.6 Drug sensitivity prediction

**Figure 7:**
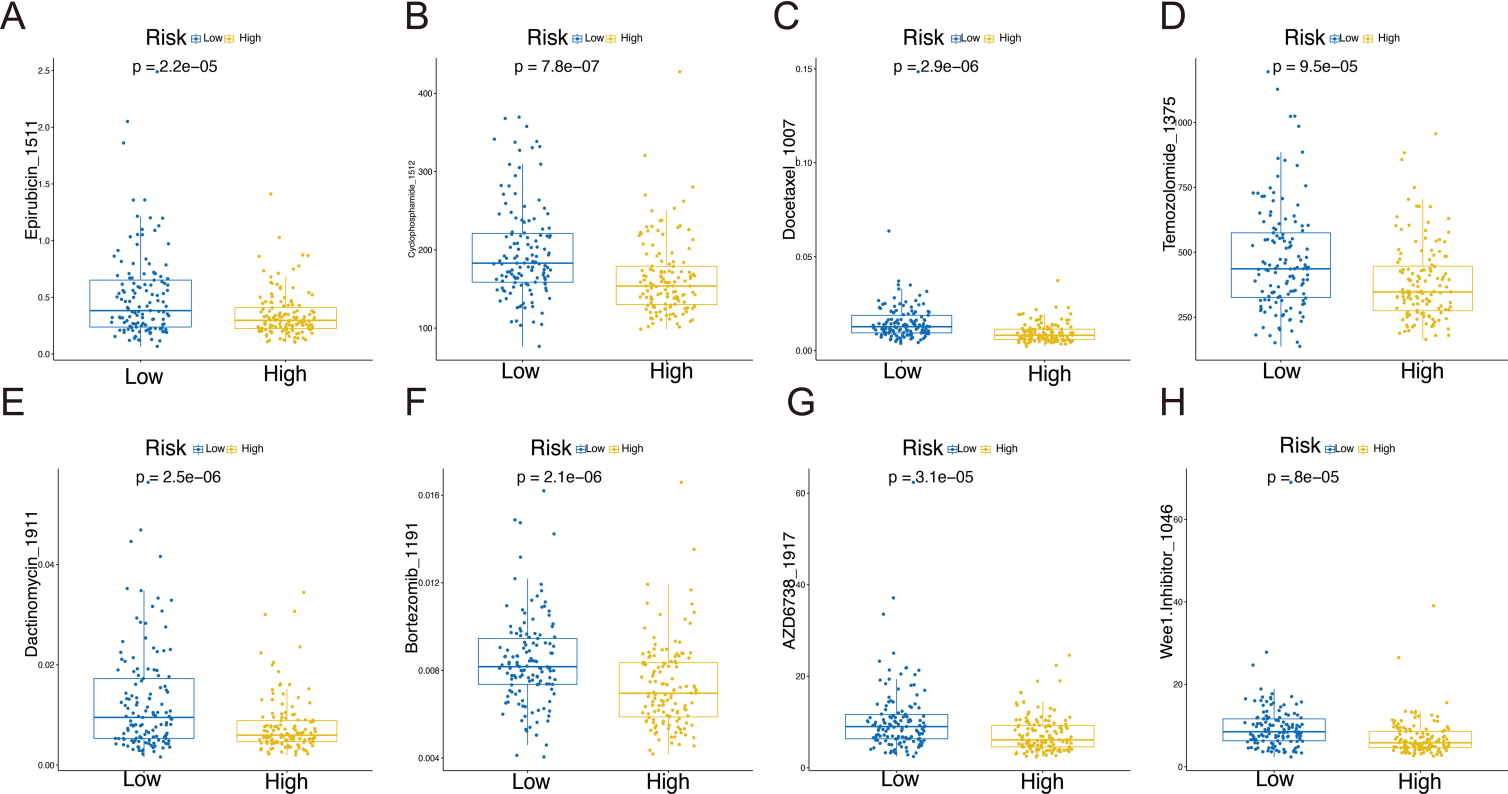
Drug sensitivity in the high-risk and low-risk groups: Epirubicin_1511 **(A)**, Cyclophosphamide _1512 **(B)**, Docetaxel _1007 **(C)**, Temozolomide _1375 **(D)**, Dactinomycin_1911 **(E)**, Bortezomib _1191 **(F)**, AZD6738_1917 **(G)**, and Wee1.Inhibitor _1046 **(H)**.

Previous studies have shown that patients treated with a combination of chemotherapy and immune-therapy or small molecule targeted therapies experience a significant improvement in survival (13). To enhance clinical treatment approaches, we employed “OncoPredict” to evaluate the drug sensitivity of STS patients and observed notable differences between the high-risk and low-risk groups. A total of 39 compounds exhibited statistically significant differences (P < 0.001) between the high-risk and low-risk groups, as summarized in **Supplementary Table 9**. The high-risk group demonstrated increased sensitivity to Epirubicin, Cyclophosphamide, Docetaxel, Temozolomide, Dactinomycin, Bortezomib, AZD6738, and Wee1.Inhibitor, underscoring the relationship between riskscore and drug responsiveness (**Figures 7A–H**).

### 3.7 Evaluation of immunotherapy response

**Figure 8:**
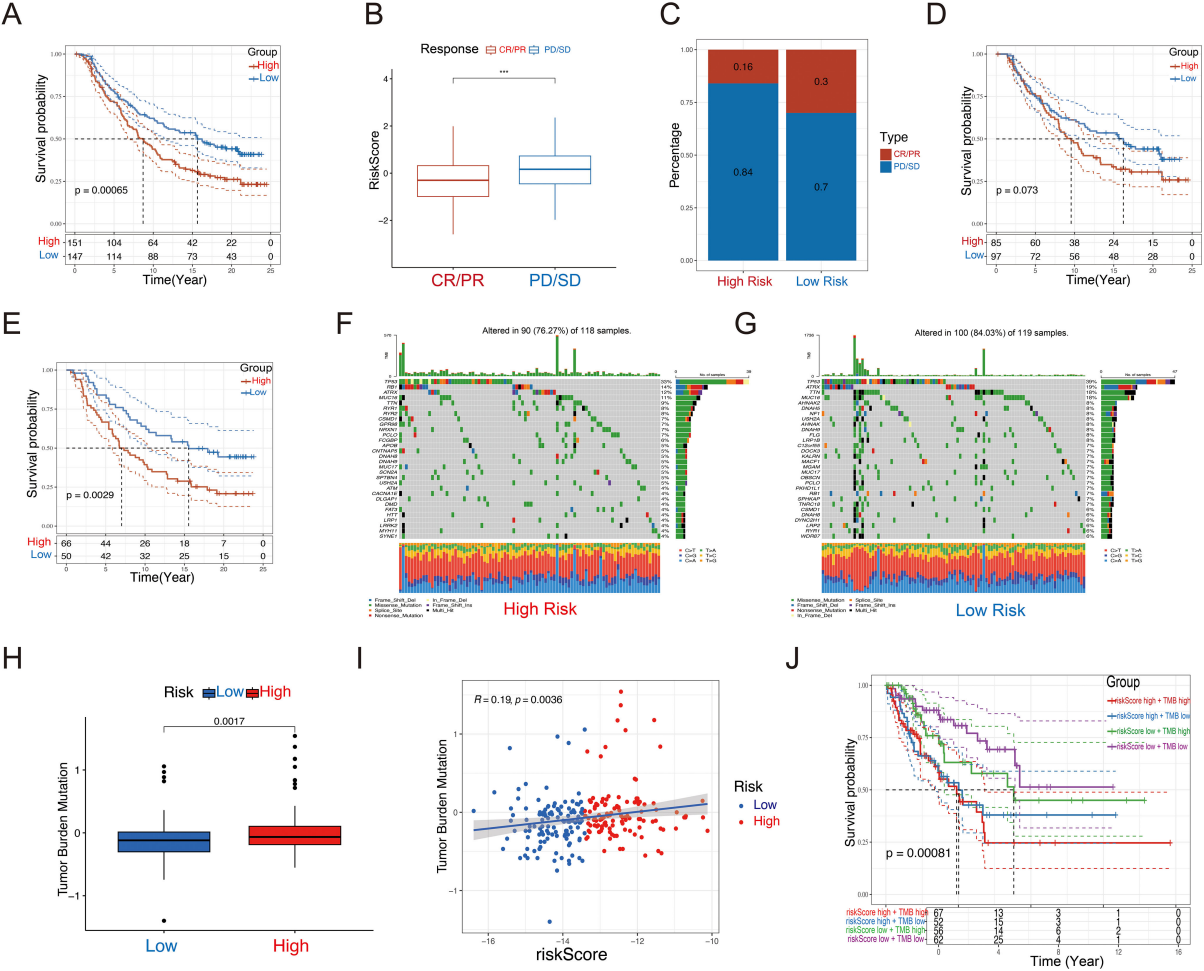
Immunotherapeutic responses and tumor mutational burden of the GRPS. **(A)** Disparities in prognosis based on riskscore classifications in the IMvigor210 cohort. **(B)** Variations in riskscores in relation to immunotherapy outcomes in the IMvigor210 dataset. **(C)** Spread of immunotherapy results across different riskscore categories within the IMvigor210 sample. **(D)** Differences in prognosis among early-stage patients based on riskscores in the IMvigor210 group. **(E)** Disparities in prognosis for advanced patients sorted by riskscores in the IMvigor210 set. **(F, G)** Waterfall plot of 12 genes with different mutation frequencies between the high-risk **(F)** and low-risk groups **(G)**. **(H)** Box diagram of TMB differences in high-risk and low-risk groups. **(I)** Correlation between Riskscore and TMB. **(J)** KM survival curve analysis of patients with diverse combinations of riskscores and TMB in TCGA cohort. ***P < 0.001.

Immunotherapy has become a vital strategy in cancer treatment, providing significant survival advantages. To explore the prognostic significance of our risk features in relation to Immunotherapy response, we examined the IMvigor210 dataset. In this cohort, patients showed varied responses to PD-L1 receptor blockade, including complete response (CR), partial response (PR), stable disease (SD), and progressive disease (PD). Remarkably, patients classified as the low-risk group demonstrated considerable clinical benefit, exhibiting a significantly extended overall survival than those in the high-risk group (**Figure 8A**, P =0.00065). In comparison to CR/PR patients, those with PD/SD had higher riskscores (**Figure 8B**). The high-risk group contained a larger proportion of PD/SD patients compared to the low-risk group (**Figure 8C**). Survival differences between risk groups varied across disease stages. While no notable difference was observed in early-stage patients (I+II, **Figure 8D**, P = 0.073), a significant distinction was evident in late-stage patients (III+IV, **Figure 8E**, P = 0.0029), underscoring the sensitivity of the riskscore in advanced disease stages.

### 3.8 Tumor mutation burden and copy number variation analysis

We assessed the TMB and CNV in the high- and low-risk groups in the TCGA-SARC cohort. The waterfall plot illustrates the mutation frequencies of the top 30 genes in each group. The high-risk group exhibited a higher mutation frequency compared to the low-risk group (**Figures 8F, G**). Furthermore, the high-risk group displayed a higher TMB score, which showed a positive correlation with the riskscore (**Figures 8H, I**). We performed KM survival analysis based on the riskscore and TMB score, and we found that the low TMB and low-risk score group exhibited the best survival status, with a longer survival time (**Figure 8J**).

### 3.9 Functional analysis of model genes and high/low-risk groups

**Figure 9:**
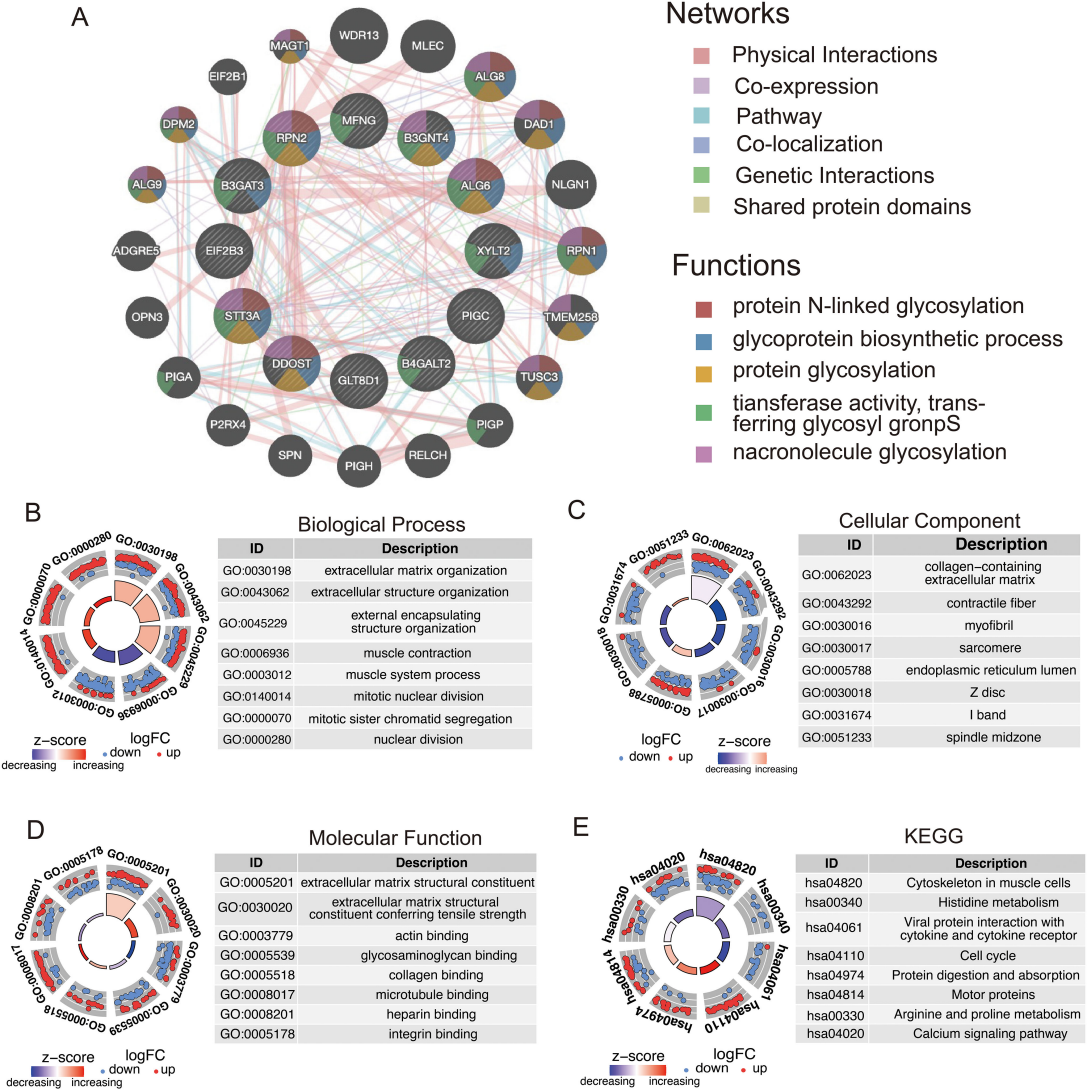
Functional analysis of model genes in relation to high- and low-risk groups. **(A)** Co-expression network of GRGS proteins analyzed by GeneMANIA. **(B–D)** GO enrichment analysis of model genes, including biological processes (BP), cellular components (CC), and molecular functions (MF). **(E)** KEGG pathways enrichment analysis for genes differentially expressed in high- and low-risk groups.

Using the GeneMANIA database, we performed an in-depth analysis of the 12 model genes to predict their potential interaction mechanisms (**Figure 9A**). These genes are participating in various processes, including protein N-linked glycosylation, glycoprotein biosynthetic processes, and protein glycosylation. These findings suggest that these genes in GRPS may impact the progression of STS by modulating metabolic signaling pathways. GO enrichment analysis revealed several associated pathways, such as extracellular matrix organization, glycosaminoglycan binding, and endoplasmic reticulum lumen, along with other relevant GO terms (**Figures 9B–D**). KEGG pathway analysis highlighted pathways associated with the cell cycle, protein digestion and absorption, as well as the calcium signaling pathway (**Figure 9E**). These results underscore the molecular and functional characteristics of STS patients, offering valuable insights into potential therapeutic targets and the underlying mechanisms.

### 3.10 Experimental validation

**Figure 10:**
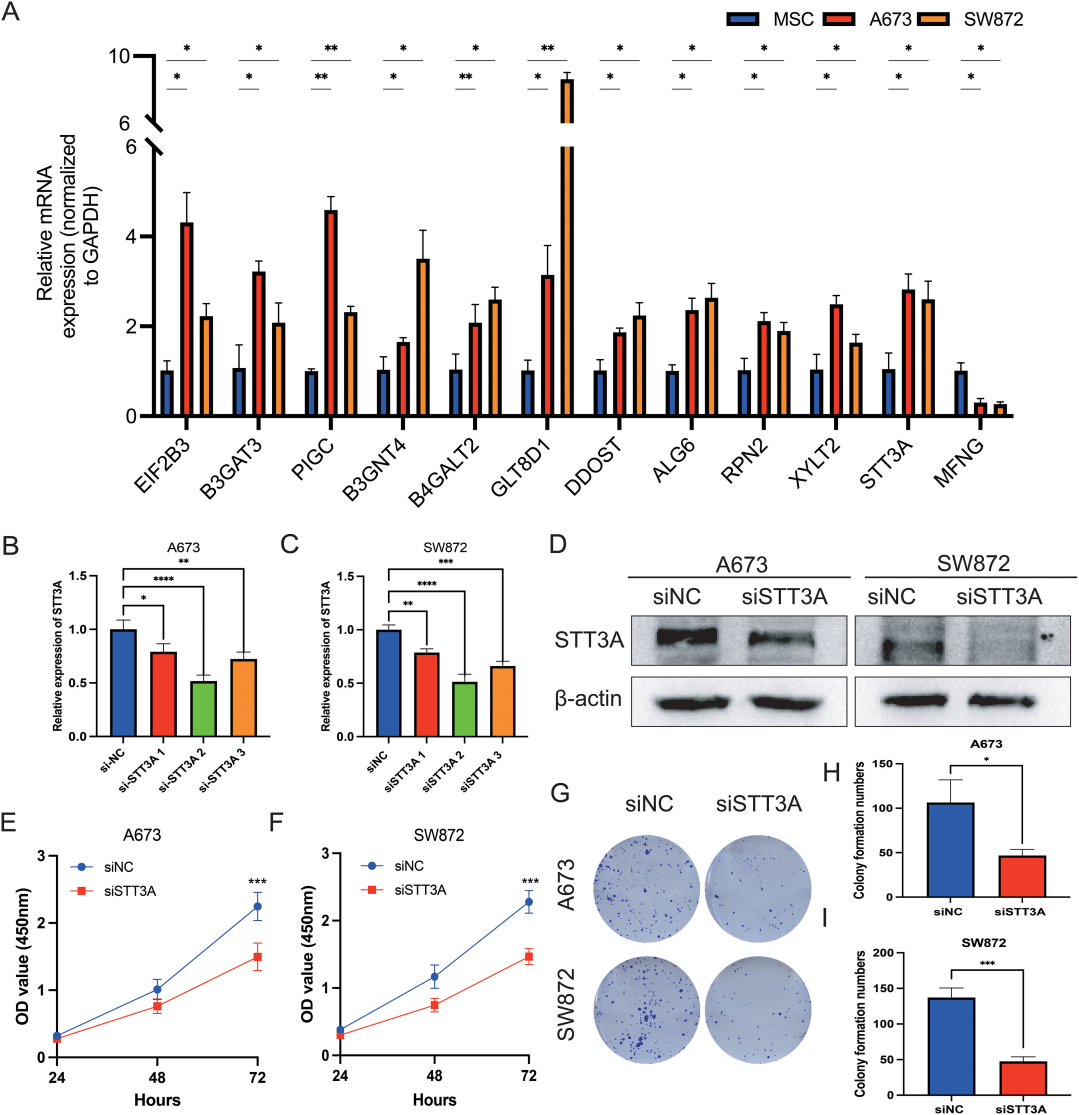
qPCR validation of 12 GRGS model genes and STT3A regulates the proliferation and viability of STS cells. **(A)** Relative mRNA expression levels of the 12 genes included in the GRGS signature were measured via qPCR. mRNA expression profiles were assessed in A673 and SW872 STS cell lines, using MSCs as non-tumor controls. **(B, C)** The expression levels by qPCR of STT3A in STS cells after STT3A knockdown. **(D)** The expression levels by Western Blotting of STT3A in STS cells after STT3A knockdown. The viability capacity of STS cells after STT3A knockdown was assessed using the CCK-8 assay **(E, F)**. The colony performed assay **(G–I)** was conducted to assess the proliferative potential of STS cells. *P < 0.05; **P < 0.01; ***P < 0.001; ****P < 0.0001.

**Figure 11:**
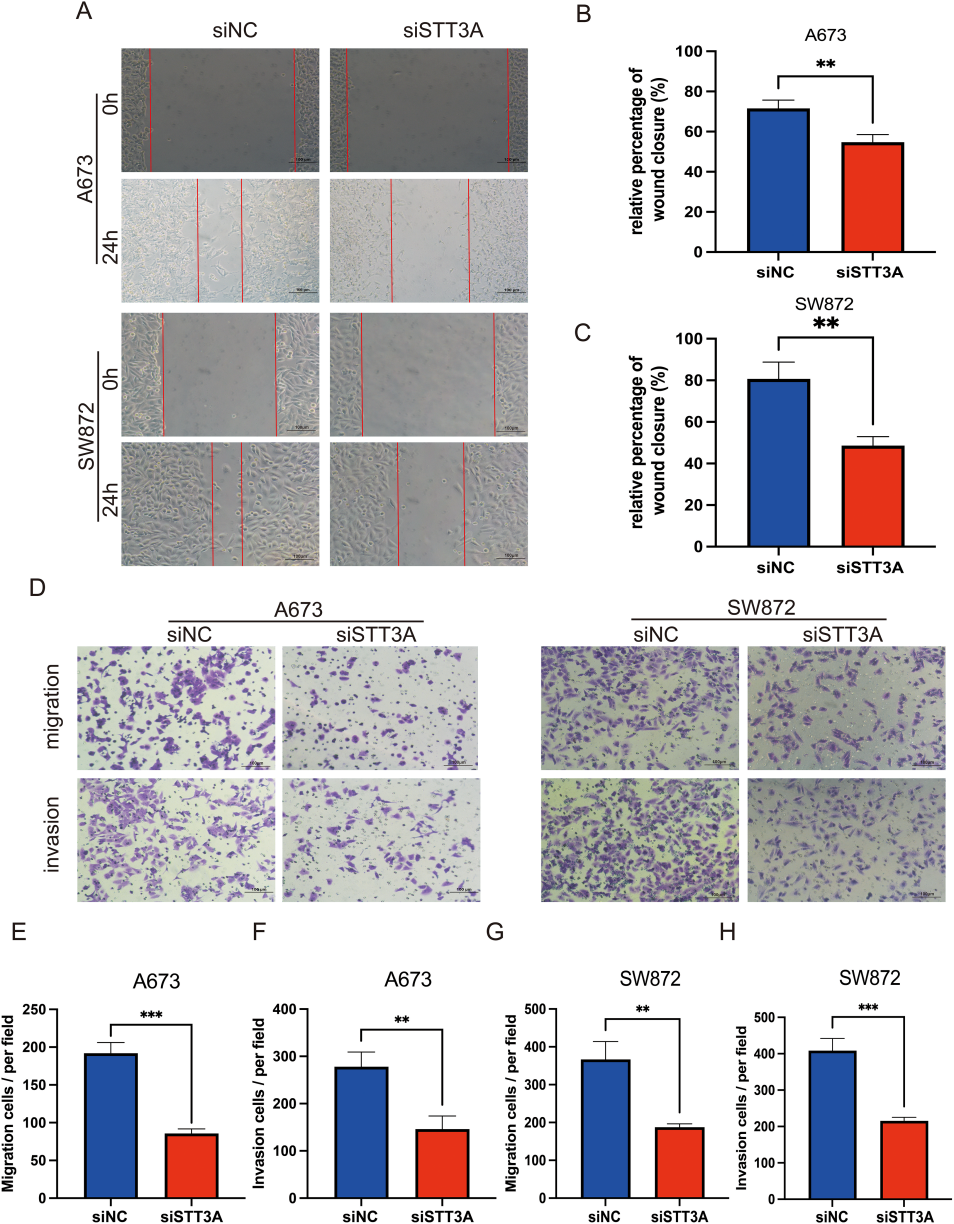
STT3A knockdown inhibits the migration and invasion abilities of STS cells. Wound healing assay **(A–C)** was performed to assess the migration abilities of STS cells after STT3A silencing. Transwell assay **(D–H)** were conducted to assess the migration and invasion abilities of STS cells by Transwell assay. **(D)** Representative microscopic images of the Transwell assay. **(E, F)** Quantification of migration and invasion in A673 cells. **(G, H)** Quantification of migration and invasion in SW872 cells. Data are presented as mean ± SD. **P < 0.01; ***P < 0.001.

Among the 12 candidate genes assessed via RT-qPCR in MSC, A673, and SW872 cells (**Figure 10A**), several—including *STT3A*—showed markedly elevated expression in STS cell lines. Based on both its transcript abundance and bioinformatic significance, we selected *STT3A* for further *in vitro* functional validation to explore its mechanistic role in STS progression. To confirm the function of *STT3A* in the STS progression, we conducted a series of functional assays. siRNA targeting *STT3A* was transfected into human rhabdomyosarcoma cell line A673 and human liposarcoma cell line SW872 to knockdown *STT3A*, and the gene silencing effect was confirmed by qPCR, assessing the mRNA expression levels in the knockdown group compared to siNC group. (**Figures 10B, C**). We also confirmed the knockdown effect of *STT3A* in STS cells using Western blotting. The Western blotting bands revealed a significant reduction in STT3A protein expression in the siSTT3A group compared to siNC group (**Figure 10D**). CCK-8 assays were conducted to examine the role of STT3A in cell viability in STS cells (**Figures 10E, F**). The findings showed that silencing *STT3A* inhibited the viability of STS cells. Additionally, colony formation assays showed that knockdown of *STT3A* suppressed the proliferative capacity of STS cells (**Figures 10G–I**). To investigate the role of *STT3A* in cell migration and invasion, we conducted wound healing assay (**Figures 11A–C**) and transwell assay (**Figures 11D–H**). The findings revealed that silencing *STT3A* markedly diminished the migration and invasion capabilities of STS cells. Overall, these results provide strong evidence that *STT3A* plays an important part in the proliferation and migration of STS cells.

## 4 Discussion

Soft tissue sarcoma is a highly malignant tumor with a rising global incidence, posing a significant public health concern (1). Traditional STS research has primarily focused on identifying recurrent gene mutations, characterizing oncogenic signaling pathways such as PI3K/AKT/mTOR and RAS/MAPK, and exploring immune checkpoint expression and tumor-infiltrating lymphocytes (14, 15). However, post-translational modifications—particularly glycosylation—have received comparatively little attention, despite their known roles in tumor progression, immune evasion, and therapeutic resistance. Therefore, we conducted a comprehensive analysis using machine learning and multiple bioinformatics approaches focused on Glycosylation alternation.

The early clinical and histological variability of STS makes its diagnosis particularly difficult, stressing the urgent need for new and effective techniques to diagnose and improve the prognosis (2). Altered glycosylation, resulting from changes in the expression or activity of glycosyltransferases, is commonly seen in tumor cells and is closely linked to cancer progression and metastasis (7). Therefore, changes in glycosylation patterns are commonly used for cancer diagnosis and prognostic evaluation.

To construct a glycosylation-related prognostic signature, we adopted a comprehensive 101-machine learning framework, with the LASSO combined with plsRcox identified as the optimal model. This integrated approach is highly flexible and well-suited for analyzing complex, high-dimensional datasets, enabling the effective selection of key glycosylation-associated prognostic features. By aggregating predictions from multiple algorithms, it mitigates bias and overfitting inherent in single-model strategies, thereby enhancing the generalizability and robustness of GRPS across independent datasets. This is crucial for validating the effectiveness and stability of GRPS across diverse clinical cohorts.

This signature was established by integrating 12 genes—*RPN2*, *ALG6*, *XYLT2*, *DDOST*, *MFNG*, *PIGC*, *EIF2B3*, *B3GAT3*, *B3GNT4*, *B4GALT2*, *GLT8D1*, and *STT3A*—all of which are functionally linked to glycosylation pathways. In this study, each gene exhibits distinct biochemical roles and molecular characteristics. The following outlines their key functional characteristics.

*RPN2* is a component of the N-oligosaccharide transferase complex and serves as a precondition for the development of many malignancies (16). Previous studies have demonstrated that *RPN2*-mediated glycosylation of CD63 correlates with the malignancy of breast cancer cells. Inhibition of *RPN2* expression was shown to reduce CD63 glycosylation and disrupt its proper localization. Moreover, silencing CD63 led to decreased drug resistance and invasive capabilities in breast cancer cells, highlighting its contribution to tumor aggressiveness and potential therapeutic targeting (17). In addition, another study reported that downregulation of the *RPN2* gene effectively induced apoptosis in docetaxel-resistant breast cancer cells (MCF7–ADR cells). *RPN2*-specific siRNA significantly suppressed tumor growth, suggesting that RPN2 may serve as a novel therapeutic target for overcoming drug resistance in breast cancer (16). Another study suggests that *RPN2* may play a role in immune regulation beyond its canonical function in N-glycosylation. In rheumatoid arthritis, *RPN2* expression is significantly upregulated in peripheral blood mononuclear cells and PHA-activated primary T lymphocytes, indicating its involvement in immune cell activation, proliferation, and apoptosis (18).

*ALG6* encodes a protein that is a member of the ALG6/ALG8 glucosyltransferase family. This enzyme facilitates the attachment of the initial glucose residue to the expanding lipid-linked oligosaccharide precursor in the N-linked glycosylation pathway. Diseases associated with *ALG6* are primarily linked to congenital disorders of glycosylation (19).

Although its role in tumors has been less studied, research by Wei, Qingyi et al. suggests that variants in *ALG6*, as part of the glycosylation pathway, could serve as potential prognostic biomarkers for cutaneous melanoma (20). Nevertheless, the specific mechanistic role of ALG6 in tumor biology remains to be elucidated and warrants further investigation.

*XYLT2* encodes xylosyltransferase II, which catalyzes the transfer of xylose from UDP-xylose to specific serine residues in proteoglycan core proteins, thus playing a key role in the synthesis of chondroitin and heparan sulfate proteoglycan (21). Previous research has shown that *XYLT2* plays a critical role in adipose tissue homeostasis by regulating glycosaminoglycan (GAG) synthesis. *XYLT2* deficiency disrupts GAG production in the extracellular matrix, resulting in impaired adipocyte differentiation, increased inflammation, and metabolic imbalances such as insulin resistance and glucose intolerance (22). This gene’s genetic variations have been linked to an increased risk of diabetic nephropathy, osteoarthritis, and elastotic pseudoxanthoma (23). Although its role in cancer remains underexplored, recent studies suggest that elevated *XYLT2* expression may correlate with poor prognosis in liver cancer. Within the EGOT–miR-32-5p–XYLT2 axis identified in our ceRNA network, *XYLT2* may contribute to hepatocarcinogenesis by modulating matrix composition and tumor–stromal interactions, warranting further mechanistic investigation (24).

The *DDOST* gene encodes a subunit of the oligosaccharide transferase complex, which is essential for glycosylating asparagine residues in newly synthesized polypeptide (25). Recent research has identified *DDOST* as a key gene involved in congenital disorders of glycosylation. Mutations in *DDOST* impair the N-glycosylation process by disrupting the function of the oligosaccharyltransferase complex, which transfers sugar chains to newly synthesized proteins in the endoplasmic reticulum. This deficiency can lead to reduced glycosylation levels and broad organ dysfunction (26). Previous studies have demonstrated that *DDOST* plays a critical role in regulating immune responses. As a component of the oligosaccharyltransferase complex, *DDOST* mediates N-glycosylation of MITA (also known as STING), which is essential for MITA oligomerization and its immune function during DNA viral infection. In mouse models, increased expression of *DDOST* enhanced local immune responses against HSV-1 and prolonged survival in mice with HSV encephalitis. These findings highlight the importance of *DDOST* in innate immune activation and offer new insights into the pathogenesis and potential treatment strategies for virus-related diseases (27). Increased expression of *DDOST* is associated with poor prognosis and immune infiltration in hepatocellular carcinoma, colon cancer, and glioma (28–30). Given its dual roles in glycosylation and immune regulation, *DDOST* represents a promising candidate for future studies in tumor progression and therapeutic resistance, particularly in soft tissue sarcomas where immune modulation and glycosylation dynamics remain underexplored.

*MFNG* is a glycosyltransferase belonging to the Fringe family, primarily involved in the O-glycosylation of the Notch receptor. It catalyzes the addition of N-acetylglucosamine (GlcNAc) to O-linked fucose residues on Notch, thereby modulating the receptor’s affinity for different ligands such as Delta and Jagged. This glycosylation step fine-tunes Notch signaling activity, which is essential for regulating cell differentiation, proliferation, and apoptosis (31). *MFNG* also plays a critical role in immune regulation. Studies have shown that *MFNG* is involved in the development of marginal zone B cells and T cell function (32). Its expression is closely associated with immune cell infiltration in the tumor microenvironment, and in certain cancers, high *MFNG* expression correlates with immune modulation and clinical prognosis (31). Through its regulation of Notch signaling, *MFNG* influences immune cell differentiation and activity, suggesting potential relevance in autoimmune diseases and immunotherapy.

*PIGC* encodes a subunit of the GPI-GlcNAc transferase complex, which is essential for the biosynthesis of glycosylphosphatidylinositol (GPI)-anchored proteins (33). *PIGC* enhances hepatocellular carcinoma cell proliferation and migration by modulating the cell cycle, with its overexpression linked to reduced survival rates in liver cancer patient (34). A study found mutations in *PIGC* in the pancreatic ductal adenocarcinoma cell line AsPC-1, and these mutations enhanced the motility of cancer cell (35).

*EIF2B3* functions as the gamma subunit of the eukaryotic translation initiation factor 2B (eIF2B) complex, which plays a key role in regulating protein synthesis during cellular stress. While it is not directly implicated in glycosylation, its involvement in endoplasmic reticulum stress response and protein folding processes suggests an indirect connection to glycosylation pathways within the ER (Endoplasmic reticulum) *(* 36). Notably, ER stress has emerged as a key regulator of tumor progression and immune evasion, as it can reshape the functional landscape of infiltrating immune cells and impair antitumor immunity within the tumor microenvironment (37, 38). Given these links, *EIF2B3* may contribute to the coordination between translational control and post-translational modifications such as glycosylation, thereby influencing tumor progression and immune microenvironment dynamics in STS.

Glycosyltransferases—including *B3GAT3, B3GNT4*, and *B4GALT2*—play distinct yet interconnected roles in glycan biosynthesis, which can influence tumor biology. *B3GAT3* catalyzes the final step in the assembly of the common tetrasaccharide linker region of proteoglycans, and its elevated expression in osteosarcoma and hepatocellular carcinoma has been associated with poor prognosis (39). Although *B3GNT4* is not well characterized in oncology, it is involved in the extension of poly-N-acetyllactosamine chains—a glycosylation modification known to influence cell adhesion and immune surveillance. Given the limited evidence regarding its role in cancer, further investigation is warranted to elucidate its potential impact on tumor progression (40). *B4GALT2*, which builds key β1,4-galactosylated structures in N- and O-linked glycans, has demonstrated increased expression in ovarian cancer cells, indicating a role in tumor-associated glycosylation remodeling (41, 42). *B4GALT2* has been reported to be involved in the immune exclusion process in lung adenocarcinoma, with its expression level negatively correlated with CD8^+^ T cell infiltration, and it may affect the immune therapy response by regulating the PD-1/PD-L1 pathway (43). Although each enzyme operates at a distinct point in the glycosylation pathway, their combined activities contribute to the structural diversity of glycans that mediate cell signaling, immune interactions, and extracellular matrix dynamics.

*GLT8D1*, which is a Golgi-localized enzyme, belongs to the glycosyltransferase family and is implicated in glycan biosynthesis in mammals (44). Mutations in *GLT8D1* have been identified in patients with several sarcomas, suggesting a potential role in tumorigenesis (45). Emerging evidence indicates that *GLT8D1* functions as an oncogene in head and neck squamous cell carcinoma, glioma and melanoma (46–48). *GLT8D1* promotes glioma progression by stabilizing CD133 via hypoxia-induced N-linked glycosylation, thereby supporting glioma stem cell maintenance and activating Wnt/β-catenin–dependent tumor growth; its elevated expression is closely associated with higher tumor grade and poor prognosis, underscoring its potential as a therapeutic target (44, 48).

*STT3A* encodes the catalytic subunit of the oligosaccharyltransferase complex, localized to the endoplasmic reticulum, and plays an essential role in co-translational N-linked glycosylation by transferring oligosaccharide chains from dolichol-linked oligosaccharides to specific asparagine residues on nascent polypeptides (49). This glycosylation process is critical for proper protein folding, immune recognition, and tumor antigen presentation. Previous studies have shown that *STT3A* is recruited by phosphorylated PD-L1 under IL-6-JAK1 signaling and mediates its glycosylation, enhancing PD-L1 stability and facilitating tumor immune escape in hepatocellular carcinoma (50, 51). Also, studies showed that STT3A contributes to tumor immune escape by mediating the N-glycosylation of PD-L1, which enhances PD-L1 stability and surface expression, thereby suppressing CD8^+^ T cell–mediated antitumor responses (52). These mechanistic insights suggest STT3A’s broader role in modulating immune checkpoints and glycosylation-dependent oncogenic processes.

In our study, *STT3A* was consistently upregulated in the high-risk STS subgroup and showed strong associations with poor prognosis, as evidenced by Kaplan-Meier survival curve and ROC analysis. Notably, *STT3A* was among the most immunologically enriched genes in ESTIMATE analysis, correlating with reduced cytolytic activity and increased infiltration of immunosuppressive cell types such as regulatory T cells and M2 macrophages. These findings suggest that *STT3A* may contribute to the formation of an immunologically “cold” tumor microenvironment that compromises anti-tumor immunity and impairs response to immune checkpoint inhibitors. Furthermore, functional validation experiments confirmed its oncogenic role by demonstrating enhanced proliferation and migration in STS cell lines, supporting its potential as a diagnostic and therapeutic target.

In this study, we developed a prognostic model based exclusively on 12 glycosylation-related genes, which may actively contribute to the progression of soft tissue sarcoma through glycosylation-mediated mechanisms. Patients classified into the high-risk group by the GRPS consistently exhibited poorer outcomes across all cohorts. The high AUC in the ROC analysis further emphasizes the diagnostic accuracy of GRPS.

To facilitate clinical application of the GRPS, we incorporated patient variables such as age, sex, margin status, and metastasis status to construct a nomogram for individualized risk assessment. This tool supports risk stratification and informs decisions on follow-up intensity, treatment selection, and timing of adjuvant therapies. High GRPS scores, often associated with immune suppression or therapy resistance, may help identify patients who could benefit from combination immunotherapy or targeted approaches. For broader implementation, prospective validation in diverse, multicenter cohorts is essential to confirm reproducibility. Integrating GRPS with conventional clinicopathological factors may improve its predictive accuracy. Additionally, developing user-friendly platforms—such as web-based calculators or tools integrated into electronic medical record systems—could facilitate real-time clinical application. Beyond prognostic evaluation, GRPS may also contribute to understanding the heterogeneity of soft tissue sarcoma and optimizing clinical trial design.

Next, we further assessed the clinical pathological features and prognostic of STS, encompassing the infiltration status of immune cells, CNV, and TMB. Glycosylation plays a more significant part in immune-related regulation and immunity against tumors. Crucial glycan-binding proteins, such as selectins, monogalactosyl and galectin, play a vital role as coordinators in modulating immune responses during tumor metastasis (53). The analysis of immune cell infiltration patterns provides insights into the tumor microenvironment. What’s more, the correlation between GRPS riskscore and tumor microenvironment scores underscores the intricate relationship between glycosylation and immune responses in STS. The low-risk group showed enrichment in various immune-related functions, such as antigen-presenting cell co-stimulation, checkpoint activity, and cytolytic activity. Higher immune cell infiltration in the low-risk group may contribute to their improved prognosis by fostering a more immunologically active tumor microenvironment capable of sustaining effective anti-tumor responses. This observation aligns with previous studies demonstrating that tumor-infiltrating immune cells, particularly CD8^+^ T cells and M1 macrophages, are associated with favorable clinical outcomes across multiple cancer type (54). Moreover, a more active immune landscape has been linked to enhanced responsiveness to immune checkpoint inhibitors (ICIs), as shown in melanoma and non-small cell lung cancer (55).

In immune checkpoint analysis, we found a close correlation between tumor glycosylation and the expression of immune checkpoints. Previous studies have shown that individuals with high PD-L1 and PD-1 levels are more responsive to immunosuppressive therapies (56). The study revealed that PD-L1, BTLA, and PD-1 expression levels were significantly elevated in the low-risk group compared to the high-risk group. Drug sensitivity predictions indicated notable differences between the groups, with the high-risk group exhibiting increased drug sensitivity. We hypothesize that the riskscore can effectively predict patient responses to immunotherapy, such as anti-PD-1/PD-L1 and anti-CTLA-4 treatments, with the low-risk group being more likely to benefit from immune checkpoint inhibitor therapy in STS.

Mutation analysis revealed an intriguing interaction between TMB and GRPS in soft tissue sarcoma. The combined stratification based on TMB and GRPS identified distinct prognostic subgroups, with the low-TMB/high-GRPS group exhibiting the poorest outcomes. This finding highlights a potential interplay between tumor mutational burden and glycosyltransferase-related processes in shaping disease progression in soft tissue sarcoma. Many glycosylation-related genes are known to be associated with congenital disorders, and their mutations in cancer may contribute to altered cell signaling, immune evasion, or changes in tumor microenvironment dynamics (57, 58). A recent study found that the allosteric mutation H351Q in the β-glucuronidase (GUSB) gene can significantly promote tumor progression in head and neck squamous cell carcinoma (HNSCC). This mutation causes GUSB protein to be retained in the endoplasmic reticulum, enhancing the stability and transcription of the STT3B subunit, thereby promoting aberrant N-glycosylation of PD-L1, which contributes to tumor immune evasion (59).

Despite the promising performance of our GRPS model, several limitations should be acknowledged. First, this study is based primarily on retrospective bioinformatics analyses using public datasets (TCGA and GEO), without validation in clinically sourced tissue samples. Second, while soft tissue sarcoma encompasses various histologic subtypes, our current analysis does not stratify risk according to these subtypes, which may influence prognostic outcomes. Third, although 12 glycosyltransferase-related genes were selected for model construction, functional validation was conducted only for *STT3A* using the A673 (a human Ewing sarcoma cell line) and SW872 (a human liposarcoma cell line) cell lines. Further experimental studies are needed to characterize the roles of the remaining model genes in STS development, progression, and immune modulation.

In summary, our study developed a nomogram incorporating glycosylation-related prognostic signatures and clinical variables, enabling the quantification of prognostic risk in STS patients and offering valuable guidance for personalized treatment decisions. Moreover, our investigation into the associations between GRPS and the tumor microenvironment, immune checkpoints, and drug sensitivity revealed that the low-risk group demonstrated more advantageous characteristics in terms of immune cell infiltration, immune checkpoint expression, and response to immune therapy. Furthermore, mutation analysis highlighted the intricate relationship between TMB and GRPS, providing a deeper insight into the mechanisms driving STS development, progression, and prognosis. These findings not only contribute to optimizing risk stratification strategies for STS patients but also provide crucial evidence for future clinical practices in precision immunotherapy.

## 5 Conclusion

This study systematically investigated the impact of glycosylation-related genes on the prognosis of soft tissue sarcoma and the regulation of the immune microenvironment. By analyzing transcriptomic data from the TCGA database, a 12 glycosylation-related genes-based prognostic signature was constructed using machine learning algorithms. We constructed a high-low risk model of soft tissue sarcoma. Patients with varying glycosylation riskscores showed notable disparities in prognosis, immune cell infiltration, and treatment response. High-risk patients exhibited lower overall survival and a more immunosuppressive tumor microenvironment, potentially leading to decreased responsiveness to immune checkpoint inhibitors. Functional validation further confirmed that silencing STT3A inhibited STS cell proliferation and migration. This study underscores the prognostic significance of glycosylation-related genes in STS, offering potential biomarkers for patient stratification and targeted treatment.

## Data Availability

The original contributions presented in the study are included in the article/**Supplementary Material**. Further inquiries can be directed to the corresponding author.

## Supplementary material

Supplementary Figure 1 NMF clustering was performed on differentially expressed GRGs. **(A)** Rank survey for nonnegative matrix factorization. **(B)** Heatmaps of consensus matrices for k=2-9.

Supplementary Figure 2 Distribution of regression coefficients in the multivariate Cox. This figure illustrates the Cox regression coefficients of the 12 genes included in the glycosylation-related prognostic signature (GRPS). Red bars indicate risk-associated genes (positive coefficients), while blue bars represent protective genes (negative coefficients). The x-axis denotes the magnitude of regression coefficients, and the y-axis lists gene names.

Supplementary Figure 3 Kaplan-Meier survival curves and ROC analyses of 12 GRPS model genes. Prognostic and predictive performance of 12 GRGS model genes via Kaplan-Meier survival and ROC curve analyses. Kaplan-Meier survival plots (top panel) show the stratification of patient groups based on high vs. low expression levels of each GRGS gene, with significant survival differences indicated by log-rank p-values. ROC curves (bottom panel) evaluate the classification power of each gene for 1-, 3-, and 5-year overall survival prediction. The AUC values quantify the sensitivity and specificity of individual genes in the GRGS signature.
