# Supplementary material for: Construction of a glycosylation-related prognostic signature for predicting prognosis, tumor microenvironment, and immune response in soft tissue sarcoma
Source: Front Oncol. 2025 Sep 2;15:1636830. doi: 10.3389/fonc.2025.1636830 (PMC12436507; doi:10.3389/fonc.2025.1636830)
Supplement: Supplementary file 1 [file Table1.docx]

**Supplementary Table 1.** 240 Glycosylation -related genes (GRGs)

| Rank | Gene Symbol | Rank | Gene Symbol | Rank | Gene Symbol | Rank | Gene Symbol |
| --- | --- | --- | --- | --- | --- | --- | --- |
| 1 | GLT8D1 | 61 | HAS2 | 121 | B3GAT2 | 181 | PIGQ |
| 2 | POMGNT2 | 62 | ST8SIA5 | 122 | ALG14 | 182 | PIGX |
| 3 | ALG1 | 63 | GYS1 | 123 | GALNT1 | 183 | PIGY |
| 4 | B4GALT2 | 64 | HAS3 | 124 | B4GALT6 | 184 | B3GALTL |
| 5 | ALG1L2 | 65 | ST8SIA1 | 125 | B3GALNT2 | 185 | PLOD3 |
| 6 | B3GNT4 | 66 | POMGNT1 | 126 | PIGA | 186 | GALNTL1 |
| 7 | FUT5 | 67 | ST3GAL3 | 127 | GXYLT2 | 187 | B3GNT1 |
| 8 | B3GALT6 | 68 | GTDC1 | 128 | B3GNT2 | 188 | GALNTL2 |
| 9 | CHPF | 69 | UGGT2 | 129 | STT3B | 189 | GALNTL4 |
| 10 | B3GAT3 | 70 | POGLUT2 | 130 | EXTL2 | 190 | GALNTL5 |
| 11 | B3GNT9 | 71 | GLT8D2 | 131 | ALG6 | 191 | GALT |
| 12 | B3GALT4 | 72 | B4GALNT2 | 132 | OGT | 192 | RPN1 |
| 13 | B3GNTL1 | 73 | B4GALNT4 | 133 | ALG13 | 193 | RPN2 |
| 14 | RFNG | 74 | ST8SIA6 | 134 | ALG10 | 194 | GGTA1 |
| 15 | ST6GALNAC2 | 75 | ST8SIA2 | 135 | ALG10B | 195 | GLA |
| 16 | XXYLT1 | 76 | ST3GAL2 | 136 | B3GALT2 | 196 | GLB1 |
| 17 | ALG3 | 77 | FUT1 | 137 | GYS2 | 197 | GLT25D1 |
| 18 | ALG1L | 78 | GBGT1 | 138 | B3GALT1 | 198 | GLT25D2 |
| 19 | ST6GALNAC6 | 79 | EXT2 | 139 | MFNG | 199 | GLT6D1 |
| 20 | ST6GALNAC4 | 80 | ABO | 140 | CSGALNACT1 | 200 | GLT8D3 |
| 21 | ST6GALNAC5 | 81 | POMT1 | 141 | PIGB | 201 | C1GALT1C1 |
| 22 | PIGV | 82 | PIGM | 142 | EOGT | 202 | CEECAM1 |
| 23 | MGAT1 | 83 | B4GALT3 | 143 | FUT9 | 203 | GALNACT1 |
| 24 | B4GALT7 | 84 | POFUT2 | 144 | EXTL3 | 204 | GYLTL1B |
| 25 | MGAT4B | 85 | GALNT2 | 145 | FUT10 | 205 | DAD1 |
| 26 | ALG8 | 86 | B4GALT1 | 146 | B4GALNT3 | 206 | DDOST |
| 27 | GCNT2 | 87 | GYG1 | 147 | C1GALT1 | 207 | DPAGT1 |
| 28 | GALNT14 | 88 | B3GNT5 | 148 | GALNT7 | 208 | KDELC1 |
| 29 | GALNT18 | 89 | ALG2 | 149 | UGCG | 209 | UGT1A6 |
| 30 | CSGALNACT2 | 90 | POGLUT1 | 150 | GCNT4 | 210 | KDELC2 |
| 31 | FUT11 | 91 | POGLUT3 | 151 | ALG11 | 211 | UGT1A8 |
| 32 | MGAT5B | 92 | STT3A | 152 | GXYLT1 | 212 | DPM2 |
| 33 | PYGM | 93 | ALG9 | 153 | GCNT7 | 213 | KTELC1 |
| 34 | CHSY1 | 94 | EXT1 | 154 | GALNT12 | 214 | UGT2A1 |
| 35 | ST6GAL1 | 95 | DPM1 | 155 | GYG2 | 215 | DPM3 |
| 36 | GALNT16 | 96 | POFUT1 | 156 | PIGZ | 216 | LARGE |
| 37 | GALNT15 | 97 | B4GALT5 | 157 | FUT4 | 217 | UGT2B10 |
| 38 | B3GAT1 | 98 | ST3GAL5 | 158 | GALNT4 | 218 | EIF2B3 |
| 39 | ST3GAL1 | 99 | B3GNT6 | 159 | ST8SIA3 | 219 | UGT2B11 |
| 40 | HAS1 | 100 | B3GNT7 | 160 | B4GALT4 | 220 | EIF2B5 |
| 41 | FUT7 | 101 | LFNG | 161 | GALNT3 | 221 | LOC152586 |
| 42 | ST3GAL4 | 102 | GCNT1 | 162 | CHPF2 | 222 | UGT2B15 |
| 43 | A4GALT | 103 | ST6GALNAC1 | 163 | GALNT11 | 223 | MAN1A1 |
| 44 | EXTL1 | 104 | GALNT6 | 164 | GALNT5 | 224 | UGT2B17 |
| 45 | XYLT2 | 105 | GALNTL6 | 165 | B3GALT5 | 225 | MAN1A2 |
| 46 | ST6GAL2 | 106 | COLGALT2 | 166 | MGAT5 | 226 | UGT2B28 |
| 47 | PYGL | 107 | A4GNT | 167 | UGGT1 | 227 | MAN1B1 |
| 48 | GALNT9 | 108 | GALNT10 | 168 | FUT8 | 228 | UGT2B4 |
| 49 | ST3GAL6 | 109 | GLT1D1 | 169 | MGAT2 | 229 | MAN1C1 |
| 50 | POMT2 | 110 | ST6GALNAC3 | 170 | A3GALT2 | 230 | UGT2B7 |
| 51 | COLGALT1 | 111 | FUT3 | 171 | NAGA | 231 | MAN2A1 |
| 52 | B3GNT3 | 112 | FUT6 | 172 | NEU1 | 232 | UGT3A1 |
| 53 | ALG12 | 113 | MGAT4A | 173 | NEU2 | 233 | FLJ21865 |
| 54 | B3GALNT1 | 114 | GALNT8 | 174 | NEU3 | 234 | MAN2A2 |
| 55 | B4GALNT1 | 115 | B3GNT8 | 175 | GAL | 235 | UGT3A2 |
| 56 | GALNT13 | 116 | GCNT3 | 176 | NEU4 | 236 | FUCA1 |
| 57 | CHSY3 | 117 | FUT2 | 177 | GALC | 237 | MANBA |
| 58 | ST8SIA4 | 118 | MGAT3 | 178 | PIGC | 238 | UGT8 |
| 59 | MGAT4C | 119 | XYLT1 | 179 | PIGH | 239 | FUCA2 |
| 60 | PYGB | 120 | ALG5 | 180 | PIGP | 240 | WBSCR17 |

**Supplementary Table 2.** Primers for qRT-PCR and small interfering RNAs

| Primers | | Sequence (5’-3’) |
| --- | --- | --- |
| STT3A | Forward | GTCTGAGCATCAGCCCACAA |
|  | Reverse | GGGCATCAGACAGGTTGCTA |
| GAPDH | Forward | GCACCGTCAAGGCTGAGAAC |
|  | Reverse | TGGTGAAGACGCCAGTGGA |
| EIF2B3 | Forward | GTGGAGGATCTCGGATGACAG |
|  | Reverse | GCTCAAGCAGGTTCAATGGGT |
| B3GAT3 | Forward | AAGGAGTCGTCTACTTTGCTGA |
|  | Reverse | GGGCATTGGGCTTATCTAACAG |
| PIGC | Forward | TCAACCTGTGACTAACACCAAGG |
|  | Reverse | GCCGGTCCACATAGTTATCAG |
| B3GNT4 | Forward | ACACAGTGTCTAGCGCCTCT |
|  | Reverse | AAGGTATCCTTGGAACAGCCT |
| MFNG | Forward | TGCTGAGTTCGACACCTTCTT |
|  | Reverse | GCCCTTGGGTTCACATAGTTG |
| GLT8D1 | Forward | AAATGCTCTCCGACATGCAGT |
|  | Reverse | TTGGAGCGAGTGTTGTGCTG |
| DDOST | Forward | ATGGGGTACTTCCGGTGTG |
|  | Reverse | GAAAAGCGAATGAGTCTCCCG |
| ALG6 | Forward | TATTCAGGTGCTGGTAAACCGC |
|  | Reverse | TGGGTAATCCAATCCCCAATACT |
| B4GALT2 | Forward | GGGCAGACTGCTGATCGAG |
|  | Reverse | CCGGTGTCTAAAGGGGATGAT |
| RPN2 | Forward | TGGCCCTGACAATCATAGCC |
|  | Reverse | GAGTCCCACGATGGAGTAGAA |
| XYLT2 | Forward | AGGTGGTACGGGCAGTAAC |
|  | Reverse | GCTCCCTGTATCTCCGTGT |
| siSTT3A 1 | Sense | GGCUGGUCAGGAUAUACAA |
|  | Anti-Sense | UUGUAUAUCCUGACCAGCC |
| siSTT3A 2 | Sense | CCUACUACAUGUGGAUCAA |
|  | Anti-Sense | UUGAUCCACAUGUAGUAGG |
| siSTT3A 3 | Sense | GUUAUGUGUUCCUGAUCAA |
|  | Anti-Sense | UUGAUCAGGAACACAUAAC |
| siNC | Sense | UUCUCCGAACGUGUCACGUTT |
|  | Anti-Sense | ACGUGACACGUUCGGAGAATT |

**Supplementary Table 3**. R package versions used in the analysis

| Package | Version | Package | Version |
| --- | --- | --- | --- |
| abind | 1.4-8 | metaBMA | 0.6.9 |
| adabag | 5 | metadat | 1.4-0 |
| ade4 | 1.7-23 | metafor | 4.8-0 |
| admisc | 0.38 | metaplus | 1.0-6 |
| afex | 1.4-1 | metapod | 1.7.0 |
| affy | 1.78.2 | methods | 4.3.2 |
| affyio | 1.70.0 | methylumi | 2.46.0 |
| affyPLM | 1.76.1 | Metrics | 0.1.4 |
| airway | 1.20.0 | mets | 1.3.6 |
| ALL | 1.42.0 | mgcv | 1.9-1 |
| alluvial | 0.1-2 | microbenchmark | 1.5.0 |
| alphavantager | 0.1.3 | mime | 0.13 |
| Amelia | 1.8.3 | minfi | 1.46.0 |
| AnnoProbe | 0.1.7 | miniUI | 0.1.2 |
| annotate | 1.78.0 | minqa | 1.2.8 |
| AnnotationDbi | 1.62.2 | misc3d | 0.9-1 |
| AnnotationFilter | 1.26.0 | mitools | 2.4 |
| AnnotationHub | 3.8.0 | mixOmics | 6.28.0 |
| anytime | 0.3.11 | mixsqp | 0.3-54 |
| ape | 5.8-1 | mixtools | 2.0.0.1 |
| aplot | 0.2.5 | mMCPcounter | 1.1.0 |
| AppliedPredictiveModeling | 1.1-7 | mnormt | 2.1.1 |
| aricode | 1.0.3 | modelbased | 0.11.2 |
| arrangements | 1.1.9 | modeldata | 1.4.0 |
| ash | 1.0-15 | modelenv | 0.2.0 |
| AsioHeaders | 1.30.2-1 | ModelMetrics | 1.2.2.2 |
| askpass | 1.2.1 | modelr | 0.1.11 |
| assertthat | 0.2.1 | modeltools | 0.2-24 |
| AUCell | 1.22.0 | mogsa | 1.34.0 |
| audio | 0.1-11 | moments | 0.14.1 |
| babelgene | 22.9 | monocle | 2.28.0 |
| backports | 1.5.0 | monocle3 | 1.3.4 |
| BART | 2.9.9 | mosaic | 1.9.1 |
| base | 4.3.2 | mosaicCore | 0.9.4.0 |
| base.rms | 1 | mosaicData | 0.20.4 |
| base64 | 2.0.2 | MOVICS | 0.99.17 |
| base64enc | 0.1-3 | mr.raps | 0.2 |
| basilisk | 1.12.1 | MRMix | 0.1.0 |
| basilisk.utils | 1.12.1 | mRMRe | 2.1.2.2 |
| batchelor | 1.18.1 | MRPRESSO | 1 |
| BayesFactor | 0.9.12-4.7 | msigdbr | 24.1.0 |
| bayesplot | 1.12.0 | multcomp | 1.4-28 |
| BayesPrism | 2.2.2 | multcompView | 0.1-10 |
| bayestestR | 0.16.0 | MultiAssayExperiment | 1.26.0 |
| BBmisc | 1.13 | multicool | 1.0.1 |
| bbmle | 1.0.25.1 | multiMiR | 1.22.0 |
| bcellViper | 1.36.0 | multtest | 2.56.0 |
| bdsmatrix | 1.3-7 | munsell | 0.5.1 |
| beachmat | 2.16.0 | mvtnorm | 1.3-3 |
| beanplot | 1.3.1 | N2R | 1.0.3 |
| beepr | 2 | namespace | 0.9.1 |
| beeswarm | 0.4.0 | naniar | 1.1.0 |
| BH | 1.87.0-1 | nanoparquet | 0.4.2 |
| bibliometrix | 5.0.1 | Nebulosa | 1.10.0 |
| bibliometrixData | 0.3.0 | network | 1.19.0 |
| biganalytics | 1.1.22 | neuralnet | 1.44.2 |
| bigassertr | 0.1.6 | NeuralNetTools | 1.5.3 |
| bigD | 0.3.1 | nleqslv | 3.3.5 |
| biglm | 0.9-3 | nlme | 3.1-168 |
| bigmemory | 4.6.4 | nloptr | 2.2.1 |
| bigmemory.sri | 0.1.8 | NMF | 0.28 |
| bigparallelr | 0.3.2 | nnet | 7.3-20 |
| bigreadr | 0.2.5 | nnls | 1.6 |
| bigstatsr | 1.6.1 | nor1mix | 1.3-3 |
| Biobase | 2.60.0 | norm | 1.0-11.1 |
| BiocBaseUtils | 1.2.0 | nortest | 1.0-4 |
| BiocFileCache | 2.8.0 | nricens | 1.6 |
| BiocGenerics | 0.46.0 | numDeriv | 2016.8-1.1 |
| BiocIO | 1.10.0 | officer | 0.6.10 |
| BiocManager | 1.30.25 | OmnipathR | 3.8.2 |
| BiocNeighbors | 1.18.0 | oncoPredict | 0.2 |
| BiocParallel | 1.34.2 | oompaBase | 3.2.9 |
| BiocSingular | 1.16.0 | oompaData | 3.1.4 |
| BiocVersion | 3.17.1 | openalexR | 2.0.1 |
| biocViews | 1.68.2 | OpenMx | 2.21.13 |
| biomaRt | 2.56.1 | openssl | 2.3.3 |
| Biostrings | 2.68.1 | openxlsx | 4.2.8 |
| bipartite | 2.21 | openxlsx2 | 1.15 |
| bit | 4.6.0 | optimParallel | 1.0-2 |
| bit64 | 4.6.0-1 | org.Hs.eg.db | 3.17.0 |
| bitops | 1.0-9 | org.Mm.eg.db | 3.17.0 |
| blob | 1.2.4 | outliers | 0.15 |
| bluster | 1.10.0 | pacman | 0.5.1 |
| bookdown | 0.43 | padr | 0.6.3 |
| boot | 1.3-31 | pagedown | 0.22 |
| bootstrap | 2019.6 | pagoda2 | 1.0.12 |
| Boruta | 8.0.0 | palette | 0.0.2 |
| brew | 1.0-10 | paletteer | 1.6.0 |
| bridgesampling | 1.1-2 | pals | 1.1 |
| brio | 1.1.5 | pamr | 1.57 |
| brms | 2.22.0 | pander | 0.6.6 |
| Brobdingnag | 1.2-9 | parallel | 4.3.2 |
| broom | 1.0.8 | parallelDist | 0.2.6 |
| BSgenome | 1.68.0 | parallelly | 1.45.0 |
| bsicons | 0.1.2 | parameters | 0.26.0 |
| bslib | 0.9.0 | parsnip | 1.3.2 |
| bumphunter | 1.42.0 | partykit | 1.2-24 |
| BWStest | 0.2.3 | patchwork | 1.3.0 |
| ca | 0.71.1 | pathview | 1.40.0 |
| cachem | 1.1.0 | pbapply | 1.7-2 |
| Cairo | 1.6-2 | pbivnorm | 0.6.0 |
| callr | 3.7.6 | pbkrtest | 0.5.4 |
| car | 3.1-3 | pbmcapply | 1.5.1 |
| carData | 3.0-5 | PBSmodelling | 2.69.3 |
| cards | 0.6.0 | pcaPP | 2.0-5 |
| caret | 7.0-1 | pdp | 0.8.2 |
| caTools | 1.18.3 | pec | 2023.04.12 |
| CCA | 1.2.2 | performance | 0.14.0 |
| CellChat | 1.6.1 | PerformanceAnalytics | 2.0.8 |
| celldex | 1.10.1 | permute | 0.9-7 |
| cellranger | 1.1.0 | phangorn | 2.12.1 |
| ceterisParibus | 0.6 | pheatmap | 1.0.12 |
| checkmate | 2.3.2 | phytools | 2.4-4 |
| chromote | 0.5.1 | pillar | 1.10.2 |
| chron | 2.3-62 | pinfsc50 | 1.3.0 |
| CIMLR | 1.0.0 | pingr | 2.0.5 |
| circlize | 0.4.16 | PINSPlus | 2.0.7 |
| circular | 0.5-1 | pixmap | 0.4-13 |
| class | 7.3-23 | pkgbuild | 1.4.8 |
| ClassDiscovery | 3.4.5 | pkgconfig | 2.0.3 |
| classInt | 0.4-11 | pkgdown | 2.1.3 |
| cli | 3.6.5 | pkgload | 1.4.0 |
| clipr | 0.8.0 | plinkbinr | 0.0.0.9000 |
| clock | 0.7.3 | plogr | 0.2.0 |
| clue | 0.3-66 | plot3D | 1.4.1 |
| cluster | 2.1.8.1 | plotly | 4.10.4 |
| clusterGeneration | 1.3.8 | plotrix | 3.8-4 |
| clusterProfiler | 4.8.3 | pls | 2.8-5 |
| clusterRepro | 0.9 | plsRcox | 1.7.7 |
| clustree | 0.5.1 | plsRglm | 1.5.1 |
| CMplot | 4.5.1 | plumber | 1.3.0 |
| cmprsk | 2.2-12 | plyr | 1.8.9 |
| CMScaller | 2.0.1 | PMCMRplus | 1.9.12 |
| coca | 1.1.0 | png | 0.1-8 |
| coda | 0.19-4.1 | pointr | 0.2.0 |
| codetools | 0.2-20 | polspline | 1.1.25 |
| coin | 1.4-3 | polyclip | 1.10-7 |
| coloc | 5.2.3 | polycor | 0.8-1 |
| colorblindcheck | 1.0.2 | polynom | 1.4-1 |
| colorspace | 2.1-1 | poorman | 0.2.7 |
| colourpicker | 1.3.0 | posterior | 1.6.1 |
| cols4all | 0.8 | ppcor | 1.1 |
| combinat | 0.0-8 | prabclus | 2.3-4 |
| ComICS | 1.0.4 | pracma | 2.4.4 |
| commonmark | 1.9.5 | praise | 1.0.0 |
| compiler | 4.3.2 | PredictABEL | 1.2-4 |
| ComplexHeatmap | 2.16.0 | preprocessCore | 1.62.1 |
| CompQuadForm | 1.4.3 | prettyGraphs | 2.2.0 |
| concatenate | 1.0.0 | prettyunits | 1.2.0 |
| config | 0.3.2 | princurve | 2.1.6 |
| confintr | 1.0.2 | prismatic | 1.1.2 |
| conflicted | 1.2.0 | pROC | 1.18.5 |
| ConsensusClusterPlus | 1.64.0 | processx | 3.8.6 |
| ConsensusTME | 0.0.1.9000 | prodlim | 2025.04.28 |
| ConsRank | 2.1.5 | profvis | 0.4.0 |
| contfrac | 1.1-12 | progeny | 1.22.0 |
| copykat | 1.1.0 | progress | 1.2.3 |
| CORElearn | 1.57.3.1 | progressr | 0.15.1 |
| corpcor | 1.6.10 | promises | 1.3.3 |
| correlation | 0.8.7 | ProtGenerics | 1.34.0 |
| corrplot | 0.95 | proto | 1.0.0 |
| COSG | 0.9.0 | proxy | 0.4-27 |
| countrycode | 1.6.1 | proxyC | 0.5.2 |
| covr | 3.6.4 | PRROC | 1.4 |
| cowplot | 1.1.3 | pRRophetic | 0.5 |
| CoxBoost | 1.5 | ps | 1.9.1 |
| coxme | 2.2-22 | pscl | 1.5.9 |
| cpp11 | 0.5.2 | psych | 2.5.3 |
| crayon | 1.5.3 | Publish | 2023.01.17 |
| credentials | 2.0.2 | pubmedR | 0.0.3 |
| crosstalk | 1.2.1 | purrr | 1.0.4 |
| curl | 6.2.3 | pyinit | 1.1.3 |
| DALEX | 2.4.3 | qlcMatrix | 0.9.8 |
| data.table | 1.17.4 | quadprog | 1.5-8 |
| data.tree | 1.1.0 | Quandl | 2.11.0 |
| datasets | 4.3.2 | quantiseqr | 1.8.0 |
| datawizard | 1.1.0 | quantmod | 0.4.27 |
| DBI | 1.2.3 | quantreg | 6.1 |
| dbplyr | 2.5.0 | questionr | 0.8.0 |
| dcurves | 0.5.0 | QuickJSR | 1.7.0 |
| DDRTree | 0.1.5 | qvalue | 2.32.0 |
| decoupleR | 2.6.0 | R.cache | 0.17.0 |
| DelayedArray | 0.26.7 | R.matlab | 3.7.0 |
| DelayedMatrixStats | 1.22.6 | R.methodsS3 | 1.8.2 |
| deldir | 2.0-4 | R.oo | 1.27.1 |
| dendextend | 1.19.0 | R.utils | 2.13.0 |
| dendsort | 0.3.4 | R6 | 2.6.1 |
| densvis | 1.10.3 | RadialMR | 1.1 |
| DEoptim | 2.2-8 | ragg | 1.4.0 |
| DEoptimR | 1.1-3-1 | rainbow | 3.8 |
| Deriv | 4.1.6 | randomcoloR | 1.1.0.1 |
| desc | 1.4.3 | randomForest | 4.7-1.2 |
| DescTools | 0.99.60 | randomForestSRC | 3.4.0 |
| DESeq2 | 1.40.2 | ranger | 0.17.0 |
| deSolve | 1.4 | RANN | 2.6.2 |
| devEMF | 4.5-1 | rappdirs | 0.3.3 |
| devtools | 2.4.5 | rARPACK | 0.11-0 |
| diagram | 1.6.5 | raster | 3.6-32 |
| DiagrammeR | 1.0.11 | rattle | 5.5.1 |
| dials | 1.4.0 | rBayesianOptimization | 1.2.1 |
| DiceDesign | 1.1 | RBGL | 1.76.0 |
| dichromat | 2.0-0.1 | rbibutils | 2.3 |
| diffobj | 0.3.6 | RCircos | 1.2.2 |
| digest | 0.6.37 | rcmdcheck | 1.4.0 |
| dimensionsR | 0.0.3 | RColorBrewer | 1.1-3 |
| diptest | 0.77-1 | Rcpp | 1.0.14 |
| dir.expiry | 1.8.0 | RcppAnnoy | 0.0.22 |
| distributional | 0.5.0 | RcppArmadillo | 14.4.3-1 |
| dlm | 1.1-6.1 | RcppEigen | 0.3.4.0.2 |
| DNAcopy | 1.74.1 | RcppGSL | 0.3.13 |
| do | 2.0.0.1 | RcppHNSW | 0.6.0 |
| doBy | 4.6.27 | RcppML | 0.3.7 |
| docopt | 0.7.2 | RcppParallel | 5.1.10 |
| doFuture | 1.0.2 | RcppProgress | 0.4.2 |
| doParallel | 1.0.17 | RcppRoll | 0.3.1 |
| doRNG | 1.8.6.2 | RcppSpdlog | 0.0.22 |
| dorothea | 1.12.0 | RcppTOML | 0.2.3 |
| DOSE | 3.26.2 | RcppZiggurat | 0.1.8 |
| dotCall64 | 1.2 | RCurl | 1.98-1.17 |
| downlit | 0.4.4 | Rdpack | 2.6.4 |
| downloader | 0.4.1 | reactable | 0.4.4 |
| dplyr | 1.1.4 | reactR | 0.6.1 |
| dqrng | 0.4.1 | readr | 2.1.5 |
| drat | 0.2.5 | readxl | 1.4.5 |
| DT | 0.33 | recipes | 1.3.1 |
| dtplyr | 1.3.1 | reformulas | 0.4.1 |
| dygraphs | 1.1.1.6 | registry | 0.5-1 |
| dynamicTreeCut | 1.63-1 | regplot | 1.1 |
| e1071 | 1.7-16 | rematch | 2.0.0 |
| easier | 1.7.1 | rematch2 | 2.1.2 |
| easierData | 1.6.0 | remotes | 2.5.0 |
| easyTCGA | 0.0.4.2000 | rentrez | 1.2.3 |
| edgeR | 3.42.4 | repr | 1.1.7 |
| effectsize | 1.0.1 | reprex | 2.1.1 |
| ElemStatLearn | 2015.6.26 | reshape | 0.8.9 |
| ellipse | 0.5.0 | reshape2 | 1.4.4 |
| ellipsis | 0.3.2 | ResidualMatrix | 1.12.0 |
| elliptic | 1.4-0 | restfulr | 0.0.15 |
| emmeans | 1.11.1 | reticulate | 1.42.0 |
| energy | 1.7-12 | rex | 1.2.1 |
| EnhancedVolcano | 1.18.0 | Rfast | 2.1.5.1 |
| enrichplot | 1.20.3 | rgl | 1.3.18 |
| ensembldb | 2.26.0 | Rgraphviz | 2.44.0 |
| entropy | 1.3.2 | rhdf5 | 2.44.0 |
| EPIC | 1.1.7 | rhdf5filters | 1.12.1 |
| estimability | 1.5.1 | Rhdf5lib | 1.22.1 |
| estimate | 1.0.13 | RhpcBLASctl | 0.23-42 |
| evaluate | 1.0.3 | Rhtslib | 2.2.0 |
| Exact | 3.3 | ridge | 3.3 |
| exactRankTests | 0.8-35 | riingo | 0.3.1 |
| ExperimentHub | 2.8.1 | rio | 1.2.3 |
| expm | 1.0-0 | riskRegression | 2025.05.20 |
| export | 0.3.0 | risksetROC | 1.0.4.1 |
| ExPosition | 2.11.0 | rJava | 1.0-11 |
| factoextra | 1.0.7 | rjson | 0.2.23 |
| FactoMineR | 2.11 | rlang | 1.1.6 |
| fansi | 1.0.6 | rlist | 0.4.6.2 |
| farver | 2.1.2 | rmarkdown | 2.29 |
| fastcluster | 1.3.0 | rmcorr | 0.7.0 |
| fastDummies | 1.7.5 | rmda | 1.6 |
| fastGHQuad | 1.0.1 | RMediation | 1.2.2 |
| fastglm | 0.0.3 | rmeta | 3 |
| fastICA | 1.2-7 | rmio | 0.4.0 |
| fastmap | 1.2.0 | Rmpfr | 1.1-0 |
| fastmatch | 1.1-6 | rms | 6.8-1 |
| fastshap | 0.1.1 | RMTstat | 0.3.1 |
| fda | 6.3.0 | rngtools | 1.5.2 |
| FDb.InfiniumMethylation.hg19 | 2.2.0 | rnndescent | 0.1.6 |
| fds | 1.8 | RobStatTM | 1.0.11 |
| ff | 4.5.2 | robustbase | 0.99-4-1 |
| fgsea | 1.26.0 | RobustRankAggreg | 1.2.1 |
| fields | 16.3.1 | ROCR | 1.0-11 |
| filelock | 1.0.3 | Rook | 1.2 |
| findPC | 1 | rootSolve | 1.8.2.4 |
| fitdistrplus | 1.2-2 | roxygen2 | 7.3.2 |
| flashClust | 1.01-2 | rpart | 4.1.24 |
| flexclust | 1.5.0 | rpart.plot | 3.1.2 |
| flexmix | 2.3-20 | rpf | 1.0.15 |
| flextable | 0.9.9 | rprojroot | 2.0.4 |
| flock | 0.7 | RPushbullet | 0.3.4 |
| FNN | 1.1.4.1 | rrcov | 1.7-7 |
| fontawesome | 0.5.3 | rsample | 1.3.0 |
| fontBitstreamVera | 0.1.1 | Rsamtools | 2.16.0 |
| fontLiberation | 0.1.0 | rscopus | 0.8.1 |
| fontquiver | 0.2.1 | RSpectra | 0.16-2 |
| forcats | 1.0.0 | RSQLite | 2.4.0 |
| foreach | 1.5.2 | rstan | 2.32.7 |
| forecast | 8.24.0 | rstanarm | 2.32.1 |
| foreign | 0.8-90 | rstantools | 2.4.0 |
| forestplot | 3.1.6 | rstatix | 0.7.2 |
| forestploter | 1.1.3 | rstudioapi | 0.17.1 |
| formatR | 1.14 | rsvd | 1.0.5 |
| Formula | 1.2-5 | rtracklayer | 1.60.1 |
| fpc | 2.2-13 | Rtsne | 0.17 |
| fracdiff | 1.5-3 | RUnit | 0.4.33 |
| fresh | 0.2.1 | rversions | 2.1.2 |
| fs | 1.6.6 | rvest | 1.0.4 |
| funModeling | 1.9.5 | rvg | 0.3.5 |
| furrr | 0.3.1 | rworldmap | 1.3-8 |
| futile.logger | 1.4.3 | s2 | 1.1.9 |
| futile.options | 1.0.1 | S4Arrays | 1.2.0 |
| future | 1.40.0 | S4Vectors | 0.38.2 |
| future.apply | 1.11.3 | S7 | 0.2.0 |
| gamm4 | 0.2-6 | sandwich | 3.1-1 |
| gargle | 1.5.2 | sargent | 1.0.1 |
| gbm | 2.2.2 | sass | 0.4.10 |
| gcrma | 2.72.0 | ScaledMatrix | 1.8.1 |
| gdata | 3.0.1 | scales | 1.4.0 |
| gdtools | 0.4.2 | scater | 1.28.0 |
| geepack | 1.3.12 | scattermore | 1.2 |
| genefilter | 1.82.1 | scatterpie | 0.2.4 |
| generics | 0.1.4 | scatterplot3d | 0.3-44 |
| GeneSwitches | 0.1.0 | sccore | 1.0.6 |
| genetics.binaRies | 0.1.1 | scHCL | 0.1.1 |
| GENIE3 | 1.26.0 | scMetabolism | 0.2.1 |
| GenomeInfoDb | 1.36.4 | scPagwas | 1.3.1 |
| GenomeInfoDbData | 1.2.10 | SCpubr | 2.0.2 |
| GenomicAlignments | 1.36.0 | scran | 1.28.2 |
| GenomicFeatures | 1.52.2 | scrime | 1.3.5 |
| GenomicRanges | 1.52.1 | scTenifoldKnk | 1.0.2 |
| GEOquery | 2.68.0 | scTenifoldNet | 1.3 |
| gert | 2.1.5 | sctransform | 0.4.2 |
| GetoptLong | 1.0.5 | scuttle | 1.12.0 |
| ggalluvial | 0.12.5 | see | 0.11.0 |
| ggbeeswarm | 0.7.2 | segmented | 2.1-4 |
| ggbiplot | 0.6.2 | selectr | 0.4-2 |
| ggcor | 0.9.8.1 | seqinr | 4.2-36 |
| ggcorrplot | 0.1.4.1 | servr | 0.32 |
| ggDCA | 1.2 | sessioninfo | 1.2.3 |
| ggdendro | 0.2.0 | set | 1.2 |
| ggdist | 3.3.3 | Seurat | 4.3.0 |
| ggExtra | 0.10.1 | SeuratDisk | 0.0.0.9021 |
| ggfittext | 0.10.2 | SeuratObject | 4.1.3 |
| ggforce | 0.4.2 | sf | 1.0-21 |
| ggformula | 0.12.0 | sfd | 0.1.0 |
| ggfortify | 0.4.17 | shades | 1.4.0 |
| ggfun | 0.1.8 | shadowtext | 0.1.4 |
| gggenes | 0.5.1 | shape | 1.4.6.1 |
| ggh4x | 0.3.1 | SHAPforxgboost | 0.1.3 |
| gghalves | 0.1.4 | shapper | 0.1.3 |
| ggmap | 4.0.1 | shapviz | 0.9.7 |
| ggnetwork | 0.5.13 | shiny | 1.10.0 |
| ggnewscale | 0.5.1 | shinycssloaders | 1.1.0 |
| ggplot2 | 3.5.2 | shinydashboard | 0.7.3 |
| ggplotify | 0.1.2 | shinydashboardPlus | 2.0.5 |
| ggpmisc | 0.6.1 | shinyjs | 2.1.0 |
| ggpol | 0.0.7 | shinystan | 2.6.0 |
| ggpp | 0.5.8-1 | shinythemes | 1.2.0 |
| ggpubr | 0.6.0 | shinyWidgets | 0.9.0 |
| ggraph | 2.2.1 | sigFeature | 1.18.0 |
| ggrastr | 1.0.2 | siggenes | 1.74.0 |
| ggrepel | 0.9.6 | SimDesign | 2.19.2 |
| ggridges | 0.5.6 | SingleCellExperiment | 1.22.0 |
| ggsankey | 0.0.99999 | SingleR | 2.2.0 |
| ggsci | 3.2.0 | singscore | 1.22.0 |
| ggside | 0.3.1 | sisal | 0.49 |
| ggsignif | 0.6.4 | sitmo | 2.0.2 |
| ggstatsplot | 0.13.1 | skimr | 2.1.5 |
| ggtext | 0.1.2 | slam | 0.1-55 |
| ggthemes | 5.1.0 | slider | 0.3.2 |
| ggtree | 3.8.2 | slingshot | 2.8.0 |
| ggvenn | 0.1.10 | sm | 2.2-6.0 |
| gh | 1.5.0 | sna | 2.8 |
| gitcreds | 0.1.2 | snakecase | 0.11.1 |
| gld | 2.6.7 | SNFtool | 2.3.1 |
| glmnet | 4.1-9 | snow | 0.4-4 |
| glmSparseNet | 1.18.0 | SnowballC | 0.7.1 |
| GlobalOptions | 0.1.2 | snowfall | 1.84-6.3 |
| globals | 0.17.0 | SOAR | 0.99-11 |
| glue | 1.8.0 | sodium | 1.4.0 |
| gmodels | 2.19.1 | sourcetools | 0.1.7-1 |
| gmp | 0.7-5 | sp | 2.2-0 |
| GO.db | 3.17.0 | spacesXYZ | 1.5-1 |
| goftest | 1.2-3 | spam | 2.11-1 |
| googleAuthR | 2.0.2 | sparcl | 1.0.4 |
| googledrive | 2.1.1 | sparkline | 2 |
| googlesheets4 | 1.1.1 | SparseArray | 1.2.3 |
| GOplot | 1.0.2 | SparseM | 1.84-2 |
| GOSemSim | 2.26.1 | sparseMatrixStats | 1.12.2 |
| gower | 1.0.2 | sparsesvd | 0.2-2 |
| GPArotation | 2025.3-1 | sparsevctrs | 0.3.4 |
| GPfit | 1.0-9 | spatial | 7.3-18 |
| gplots | 3.2.0 | spatstat.data | 3.1-6 |
| graph | 1.78.0 | spatstat.explore | 3.4-3 |
| graphics | 4.3.2 | spatstat.geom | 3.4-1 |
| graphite | 1.46.0 | spatstat.random | 3.4-1 |
| graphlayouts | 1.2.2 | spatstat.sparse | 3.1-0 |
| grDevices | 4.3.2 | spatstat.univar | 3.1-3 |
| grid | 4.3.2 | spatstat.utils | 3.1-4 |
| gridBase | 0.4-7 | spData | 2.3.4 |
| gridExtra | 2.3 | spdep | 1.3-11 |
| gridGraphics | 0.5-1 | speedglm | 0.3-5 |
| gridtext | 0.1.5 | spelling | 2.3.1 |
| grImport2 | 0.3-3 | splines | 4.3.2 |
| grr | 0.9.5 | splitstackshape | 1.4.8 |
| GSA | 1.03.3 | splus2R | 1.3-5 |
| GSEABase | 1.62.0 | sqldf | 0.4-11 |
| GseaVis | 0.1.0 | SQUAREM | 2021.1 |
| gsl | 2.1-8 | stabs | 0.6-4 |
| gson | 0.1.0 | StanHeaders | 2.32.10 |
| gsubfn | 0.7 | stargazer | 5.2.3 |
| GSVA | 1.48.3 | statmod | 1.5.0 |
| gt | 1.0.0 | statnet.common | 4.12.0 |
| gtable | 0.3.6 | stats | 4.3.2 |
| gtools | 3.9.5 | stats4 | 4.3.2 |
| gtsummary | 2.2.0 | statsExpressions | 1.7.0 |
| gwasglue | 0.0.0.9000 | STRINGdb | 2.12.1 |
| gwasglue2 | 0.0.0.9000 | stringdist | 0.9.15 |
| gwasrapidd | 0.99.18 | stringi | 1.8.7 |
| gwasvcf | 0.1.2 | stringr | 1.5.1 |
| hardhat | 1.4.1 | styler | 1.10.3 |
| harmony | 1.2.3 | SummarizedExperiment | 1.30.2 |
| hash | 2.2.6.3 | superpc | 1.12 |
| haven | 2.5.5 | SuppDists | 1.1-9.9 |
| HDF5Array | 1.28.1 | survAUC | 1.3-0 |
| hdf5r | 1.3.12 | survcomp | 1.50.0 |
| HDO.db | 0.99.1 | survey | 4.4-2 |
| hdrcde | 3.4 | survival | 3.8-3 |
| heatmap.plus | 1.3 | survivalROC | 1.0.3.1 |
| here | 1.0.1 | survivalsvm | 0.0.6 |
| HGNChelper | 0.8.15 | survminer | 0.5.0 |
| hgu133acdf | 2.18.0 | survMisc | 0.5.6 |
| hgu133plus2.db | 3.13.0 | susieR | 0.14.1 |
| hgu133plus2cdf | 2.18.0 | sva | 3.48.0 |
| highr | 0.11 | svd | 0.5.8 |
| Hmisc | 5.2-3 | svglite | 2.2.1 |
| hms | 1.1.3 | swagger | 5.17.14.1 |
| HSMMSingleCell | 1.20.0 | sys | 3.4.3 |
| htmlTable | 2.4.3 | systemfonts | 1.2.3 |
| htmltools | 0.5.8.1 | tableone | 0.13.2 |
| htmlwidgets | 1.6.4 | TCGAbiolinks | 2.28.4 |
| httpuv | 1.6.16 | TCGAbiolinksGUI.data | 1.20.0 |
| httr | 1.4.7 | TCGAmutations | 0.4.0 |
| httr2 | 1.1.2 | tcltk | 4.3.2 |
| hunspell | 3.0.6 | tensor | 1.5 |
| hypergeo | 1.2-14 | tensorA | 0.36.2.1 |
| iBreakDown | 2.1.2 | tensorflow | 2.16.0 |
| ica | 1.0-3 | terra | 1.8-54 |
| iClusterPlus | 1.36.1 | testit | 0.13 |
| ids | 1.0.1 | testthat | 3.2.3 |
| ieugwasr | 1.0.3 | textshaping | 1.0.1 |
| igraph | 2.0.3 | tfautograph | 0.3.2 |
| illuminaio | 0.42.0 | tfruns | 1.5.3 |
| iml | 0.11.4 | TH.data | 1.1-3 |
| immunedeconv | 2.1.0 | thematic | 0.1.6 |
| impute | 1.74.1 | threejs | 0.3.4 |
| infer | 1.0.8 | tibble | 3.2.1 |
| ingredients | 2.3.0 | tidygraph | 1.3.1 |
| ini | 0.3.1 | tidyHeatmap | 1.11.6 |
| inline | 0.3.21 | tidymodels | 1.3.0 |
| insight | 1.3.0 | tidyquant | 1.0.11 |
| interactiveDisplayBase | 1.38.0 | tidyr | 1.3.1 |
| InterSIM | 2.3.0 | tidyselect | 1.2.1 |
| IntNMF | 1.3.0 | tidytext | 0.4.2 |
| inum | 1.0-5 | tidytree | 0.4.6 |
| IOBR | 0.99.8 | tidyverse | 2.0.0 |
| iotools | 0.3-5 | timechange | 0.3.0 |
| ipred | 0.9-15 | timeDate | 4041.11 |
| IRanges | 2.34.1 | timereg | 2.0.6 |
| irGSEA | 3.2.2 | timeROC | 0.4 |
| irlba | 2.3.5.1 | timetk | 2.9.0 |
| isoband | 0.2.7 | tinyarray | 2.4.3 |
| ISOpureR | 1.1.3 | tinytest | 1.4.1 |
| iterators | 1.0.14 | tinytex | 0.57 |
| iterpc | 0.4.2 | tmcn | 0.2-13 |
| janeaustenr | 1.0.0 | tokenizers | 0.3.0 |
| janitor | 2.2.1 | tools | 4.3.2 |
| job | 0.3.1 | TrajectoryUtils | 1.8.0 |
| JOUSBoost | 2.1.0 | tree | 1.0-44 |
| jpeg | 0.1-11 | treeio | 1.24.3 |
| jquerylib | 0.1.4 | tricycle | 1.8.0 |
| jsonlite | 2.0.0 | triebeard | 0.4.1 |
| jstable | 1.3.12 | tseries | 0.10-58 |
| juicyjuice | 0.1.0 | tsfeatures | 1.1.1 |
| kableExtra | 1.4.0 | TSMRhelper | 0.0.9 |
| KEGGgraph | 1.60.0 | TTR | 0.24.4 |
| KEGGREST | 1.40.1 | tune | 1.3.0 |
| keras | 2.15.0 | tweenr | 2.0.3 |
| KernelKnn | 1.1.5 | TwoSampleMR | 0.5.7 |
| kernelshap | 0.7.0 | TxDb.Hsapiens.UCSC.hg19.knownGene | 3.2.2 |
| kernlab | 0.9-33 | tzdb | 0.5.0 |
| KernSmooth | 2.23-26 | UCell | 2.4.0 |
| khroma | 1.16.0 | umap | 0.2.10.0 |
| klaR | 1.7-3 | units | 0.8-7 |
| km.ci | 0.5-6 | UpSetR | 1.4.0 |
| KMsurv | 0.1-6 | urca | 1.3-4 |
| knitr | 1.5 | urlchecker | 1.0.1 |
| ks | 1.15.1 | urltools | 1.7.3 |
| kSamples | 1.2-10 | usethis | 3.1.0 |
| labeling | 0.4.3 | utf8 | 1.2.5 |
| labelled | 2.14.1 | utils | 4.3.2 |
| laeken | 0.5.3 | uuid | 1.2-1 |
| lambda.r | 1.2.4 | uwot | 0.2.3 |
| LaplacesDemon | 16.1.6 | V8 | 6.0.4 |
| lars | 1.3 | VAM | 1.1.0 |
| later | 1.4.2 | VariantAnnotation | 1.46.0 |
| lattice | 0.22-7 | vcd | 1.4-13 |
| lava | 1.8.1 | vcfR | 1.15.0 |
| lavaan | 0.6-19 | vctrs | 0.6.5 |
| lazyeval | 0.2.2 | vegan | 2.6-10 |
| leaps | 3.2 | venn | 1.12 |
| leiden | 0.4.3.1 | VennDiagram | 1.7.3 |
| leidenbase | 0.1.35 | VGAM | 1.1-13 |
| lhs | 1.2.0 | VIM | 6.2.2 |
| libcoin | 1.0-10 | vioplot | 0.5.1 |
| lifecycle | 1.0.4 | vip | 0.4.1 |
| lightgbm | 4.6.0 | vipor | 0.4.7 |
| limma | 3.56.2 | viridis | 0.6.5 |
| limSolve | 1.5.7.1 | viridisLite | 0.4.2 |
| linkET | 0.0.7.4 | visdat | 0.6.0 |
| listenv | 0.9.1 | VISION | 3.0.1 |
| litedown | 0.7 | visNetwork | 2.1.2 |
| lme4 | 1.1-37 | vroom | 1.6.5 |
| lmerTest | 3.1-3 | waiter | 0.2.5 |
| lmodel2 | 1.7-4 | waldo | 0.6.1 |
| lmom | 3.2 | warp | 0.2.1 |
| lmtest | 0.9-40 | waterfalls | 1.0.0 |
| locfit | 1.5-9.12 | wdm | 0.2.6 |
| loe | 1.1 | webshot2 | 0.1.2 |
| logger | 0.4.0 | websocket | 1.4.4 |
| logging | 0.10-108 | webutils | 1.2.2 |
| logspline | 2.1.22 | wesanderson | 0.3.7 |
| loo | 2.8.0 | WGCNA | 1.73 |
| lpSolve | 5.6.23 | whisker | 0.4.1 |
| lubridate | 1.9.4 | withr | 3.0.2 |
| lumi | 2.52.0 | wk | 0.9.4 |
| maftools | 2.16.0 | wordcloud2 | 0.2.1 |
| magick | 2.8.6 | wordspace | 0.2-8 |
| magrittr | 2.0.3 | workflows | 1.2.0 |
| mapproj | 1.2.12 | workflowsets | 1.1.1 |
| maps | 3.4.3 | writexl | 1.5.4 |
| markdown | 2 | WRS2 | 1.1-7 |
| MASS | 7.3-60.0.1 | xCell | 1.1.0 |
| mathjaxr | 1.8-0 | xfun | 0.52 |
| Matrix | 1.6-1 | xgboost | 1.7.11.1 |
| MatrixGenerics | 1.12.3 | xlsx | 0.6.5 |
| MatrixModels | 0.5-4 | xlsxjars | 0.6.1 |
| matrixStats | 1.5.0 | XML | 3.99-0.18 |
| maxstat | 0.7-26 | xml2 | 1.3.8 |
| mbend | 1.3.1 | xopen | 1.0.1 |
| mboost | 2.9-11 | xtable | 1.8-4 |
| mc2d | 0.2.1 | xts | 0.14.1 |
| mclust | 6.1.1 | XVector | 0.40.0 |
| mcmc | 0.9-8 | yaml | 2.3.10 |
| MCMCpack | 1.7-1 | yardstick | 1.3.2 |
| MCPcounter | 1.2.0 | yulab.utils | 0.2.0 |
| mediation | 4.5.0 | zeallot | 0.2.0 |
| memoise | 2.0.1 | zigg | 0.0.2 |
| memuse | 4.2-3 | zip | 2.3.3 |
| MendelianRandomization | 0.10.0 | zlibbioc | 1.46.0 |
| meta | 8.1-0 | zoo | 1.8-14 |

**Supplementary Table 4**. CCK-8 Results in A673 and SW872 Cell Lines Following STT3A Knockdown

| **Experiment** | **Group** | **obvervational index** | **Replicate 1** | **Replicate 2** | **Replicate 3** | **Mean** | **SD** |
| --- | --- | --- | --- | --- | --- | --- | --- |
| CCK-8 | siNC (A673) | OD value at 24, 48, 72h | 0.328;1.123;2.444 | 0.321;1.609;2.024 | 0.317;0.842;2.273 | 0.322;1.011;2.247 | 0.006;0.149;0.211 |
| CCK-8 | siSTT3A (A673) | OD value at 24, 48, 72h | 0.303;0.737;1.714 | 0.271;0.878;1.741 | 0.265;0.665;1.305 | 0.280;0.760;1.497 | 0.02;0.108;0.206 |
| CCK-8 | siNC (SW872) | OD value at 24, 48, 72h | 0.437;1.347;2.294 | 0.340;1.161;2.441 | 0.378;1.0;2.105 | 0.382;1.169;2.280 | 0.044;0.174;0.168 |
| CCK-8 | siSTT3A (SW872) | OD value at 24, 48, 72h | 0.3;0.839;1.597 | 0.293;0.639;1.439 | 0.313;0.763;1.366 | 0.302;0.747;1.467 | 0.01;0.101;0.108 |

**Supplementary Table 5**. Colony Formation Assay Results in A673 and SW872 Cell Lines Following STT3A Knockdown

| **Experiment** | | **Group** | **obvervational index** | **Replicate 1** | **Replicate 2** | **Replicate 3** | **Mean** | **SD** |
| --- | --- | --- | --- | --- | --- | --- | --- | --- |
| Colony assay | | siNC (A673) | Colony formation numbers | 133 | 82 | 104 | 106.333 | 25.580 |
| Colony assay | | siSTT3A (A673) | Colony formation numbers | 40 | 46 | 54 | 46.667 | 7.024 |
| Colony assay | siNC (SW872) | Colony formation numbers | 151 | 136 | 124 | 137 | 13.528 |  |
| Colony assay | siSTT3A (SW872) | Colony formation numbers | 41 | 54 | 47 | 47.333 | 6.506 |  |

**Supplementary Table 6**. Wound healing assay Results in A673 and SW872 Cell Lines Following STT3A Knockdown

| **Experiment** | **Group** | **obvervational index** | **Replicate 1** | **Replicate 2** | **Replicate 3** | **Mean** | **SD** |
| --- | --- | --- | --- | --- | --- | --- | --- |
| Wound healing assay | siNC (A673) | Scratch width at 0h and 24h; Relative percentage of  wound closure (%) | 29.8625 and 8.8978 μm  (70.2%) | 31.4 and 9.95μm (68.3 %) | 29.57 and 7.02μm (76.2%) | 71.567% | 4.124 |
| Wound healing assay | siSTT3A (A673) | Scratch width at 0h and 24h; Relative percentage of  wound closure (%) | 32.7848 and 16.2886μm (50.3%) | 29.03 and 12.72μm (56.2%) | 30.03 and 12.72μm (57.6%) | 54.7% | 3.874 |
| Wound healing assay | siNC (SW872) | Scratch width at 0h and 24h; Relative percentage of  wound closure (%) | 22.74 and 5.68 μm (75.0%) | 21.87 and 2.2 μm (89.9%) | 21.7 and 4.91μm (77.3%) | 80.733% | 8.021 |
| Wound healing assay | siSTT3A (SW872) | Scratch width at 0h and 24h; Relative percentage of  wound closure (%) | 26.79 and 13.38μm (50.0%) | 28.99 and 13.86μm (52.1%) | 26.38 and 14.82μm (43.8%) | 48.633% | 4.315 |

**Supplementary Table 7**. Transwell Migration assay Results in A673 and SW872 Cell Lines Following STT3A Knockdown

| **Experiment** | **Group** | **obvervational index** | **Replicate 1** | **Replicate 2** | | **Replicate 3** | **Mean** | **SD** |
| --- | --- | --- | --- | --- | --- | --- | --- | --- |
| Transwell Migration assay | siNC (A673) | Migration cells / per field | 190 | 207 | 179 | | 192 | 14.107 |
| Transwell Migration assay | siSTT3A (A673) | Migration cells / per field | 92 | 80 | 85 | | 85.667 | 6.028 |
| Transwell Migration assay | siNC (SW872) | Migration cells / per field | 314 | 404 | 383 | | 367 | 47.085 |
| Transwell Migration assay | siSTT3A (SW872) | Migration cells / per field | 180 | 197 | 186 | | 187.667 | 8.622 |

**Supplementary Table 8**. Transwell Invasion assay Results in A673 and SW872 Cell Lines Following STT3A Knockdown

| **Experiment** | **Group** | **obvervational index** | **Replicate 1** | **Replicate 2** | **Replicate 3** | **Mean** | **SD** |
| --- | --- | --- | --- | --- | --- | --- | --- |
| Transwell Invasion assay | siNC (A673) | Invasion cells / per field | 298 | 294 | 242 | 278 | 31.241 |
| Transwell Invasion assay | siSTT3A (A673) | Invasion cells / per field | 169 | 115 | 154 | 146 | 27.875 |
| Transwell Invasion assay | siNC (SW872) | Invasion cells / per field | 401 | 445 | 380 | 408.667 | 33.171 |
| Transwell Invasion assay | siSTT3A (SW872) | Invasion cells / per field | 205 | 224 | 218 | 215.667 | 9.713 |

**Supplementary Table 9. Drug Sensitivity Metrics and p-values for Risk-Stratified STS Samples**

| Drug | p_value | Mean_Low (μM) | Mean_High (μM) |
| --- | --- | --- | --- |
| Cisplatin_1005 | 7.55126513912309e-05 | 36.714 | 25.335 |
| Docetaxel_1007 | 2.89215246548352e-06 | 0.016 | 0.009 |
| SB216763_1025 | 3.47213686665143e-05 | 182.683 | 205.497 |
| Doramapimod_1042 | 9.65610971547565e-06 | 85.584 | 94.286 |
| Wee1.Inhibitor_1046 | 7.96931947339015e-05 | 9.758 | 6.969 |
| Obatoclax.Mesylate_1068 | 6.54878982418095e-05 | 4.695 | 3.892 |
| BI.2536_1086 | 9.15359445331829e-08 | 1.865 | 1.229 |
| MK.1775_1179 | 1.3715973769402e-05 | 2.249 | 1.649 |
| Bortezomib_1191 | 2.14469775874978e-06 | 0.008 | 0.007 |
| Fulvestrant_1200 | 6.20524536304304e-07 | 21.373 | 17.289 |
| YK.4.279_1239 | 9.49982800075362e-07 | 15.913 | 8.536 |
| BMS.345541_1249 | 4.00000195612046e-05 | 35.369 | 27.66 |
| Temozolomide_1375 | 9.54385681283765e-05 | 471.532 | 384.139 |
| Epirubicin_1511 | 2.16050929869317e-05 | 0.511 | 0.347 |
| Cyclophosphamide_1512 | 7.79356112590577e-07 | 197.104 | 163.339 |
| IRAK4_4710_1716 | 3.68658328688894e-05 | 153.696 | 133.945 |
| AZD4547_1786 | 2.33023345825477e-09 | 22.216 | 16.289 |
| Mitoxantrone_1810 | 8.70559611420819e-05 | 1.788 | 2.364 |
| Docetaxel_1819 | 1.23846206496827e-05 | 0.199 | 0.101 |
| Gallibiscoquinazole_1830 | 3.97036846608576e-07 | 15.261 | 12.76 |
| MG.132_1862 | 1.50127442370906e-10 | 0.221 | 0.184 |
| BDP.00009066_1866 | 5.66749264780182e-05 | 12.095 | 10.163 |
| Dactinomycin_1911 | 2.49984349592954e-06 | 0.013 | 0.008 |
| AZD6738_1917 | 3.13918484798395e-05 | 10.464 | 7.382 |
| Cediranib_1922 | 1.20626989728329e-08 | 10.696 | 7.9 |
| GDC0810_1925 | 2.4516927623032e-10 | 159.789 | 124.915 |
| GSK2578215A_1927 | 4.86795191457577e-11 | 156.453 | 123.019 |
| I.BRD9_1928 | 7.36720880019956e-07 | 92.41 | 73.592 |
| Telomerase.Inhibitor.IX_1930 | 3.32389251458425e-12 | 2.131 | 1.41 |
| MIRA.1_1931 | 4.60409556081475e-07 | 268.977 | 211.859 |
| NVP.ADW742_1932 | 1.51034612471227e-07 | 19.56 | 14.636 |
| P22077_1933 | 3.21566472862801e-08 | 108.31 | 80.441 |
| Savolitinib_1936 | 1.37756905117292e-06 | 15.579 | 12.821 |
| UMI.77_1939 | 1.44838503653084e-07 | 19.696 | 13.713 |
| Sepantronium.bromide_1941 | 2.01440954982457e-09 | 0.02 | 0.013 |
| MIM1_1996 | 1.06227275603863e-06 | 55.445 | 45.858 |
| BPD.00008900_1998 | 9.8129166779523e-07 | 109.182 | 87.165 |
| Pyridostatin_2044 | 2.05124460755228e-06 | 35.596 | 26.359 |
| VE821_2111 | 1.71616251711706e-06 | 77.291 | 57.215 |

**Supplementary Table 10**. Comparison of Published Prognostic Models for STS and the Present GRPS Model

| Model Type | Reference | No. of Genes | Dataset | AUC (1/3/5-year) | Biological Focus |
| --- | --- | --- | --- | --- | --- |
| immunotherapy-related genes | Mining TCGA to reveal immunotherapy-related genes for soft tissue sarcoma | 14 | TCGA-SARC | unmentioned | Immune checkpoint, TME |
| NETosis-related signatures | Deciphering the role of NETosis-related signatures in the prognosis and immunotherapy of soft-tissue sarcoma using machine learning | 17 | TCGA-SARC; GSE17118 and GSE30929 | unmentioned | NETosis |
| angiogenesis-related genes | Integrative profiling analysis reveals prognostic significance, molecular characteristics, and tumor immunity of angiogenesis-related genes in soft tissue sarcoma | 5 | TCGA-SARC;GSE21050; GSE71118 | (0.752,0.745,0.741 for TCGA; 0.627,0589, 0.531for GSE21050; 0.623,0.587, 0.553 for GSE71118) | angiogenesis-related genes |
| TLS-associated genes | Identifying specific TLS-associated genes as potential biomarkers for predicting prognosis and evaluating the efficacy of immunotherapy in soft tissue sarcoma | 2 | TCGA-SARC | (0.693;0.665 for 1- year in traning and test cohort) | tertiary lymphatic structure (TLS)-associated genes |
| Current Study (GRPS) | This study | 12 | TCGA-SARC + GSE17674 | 0.77 / 0.74 / 0.72 | Glycosylation; immunity |
