# Supplementary figures and images for: Construction of a glycosylation-related prognostic signature for predicting prognosis, tumor microenvironment, and immune response in soft tissue sarcoma
Source: Front Oncol. 2025 Sep 2;15:1636830. doi: 10.3389/fonc.2025.1636830 (PMC12436507; doi:10.3389/fonc.2025.1636830)

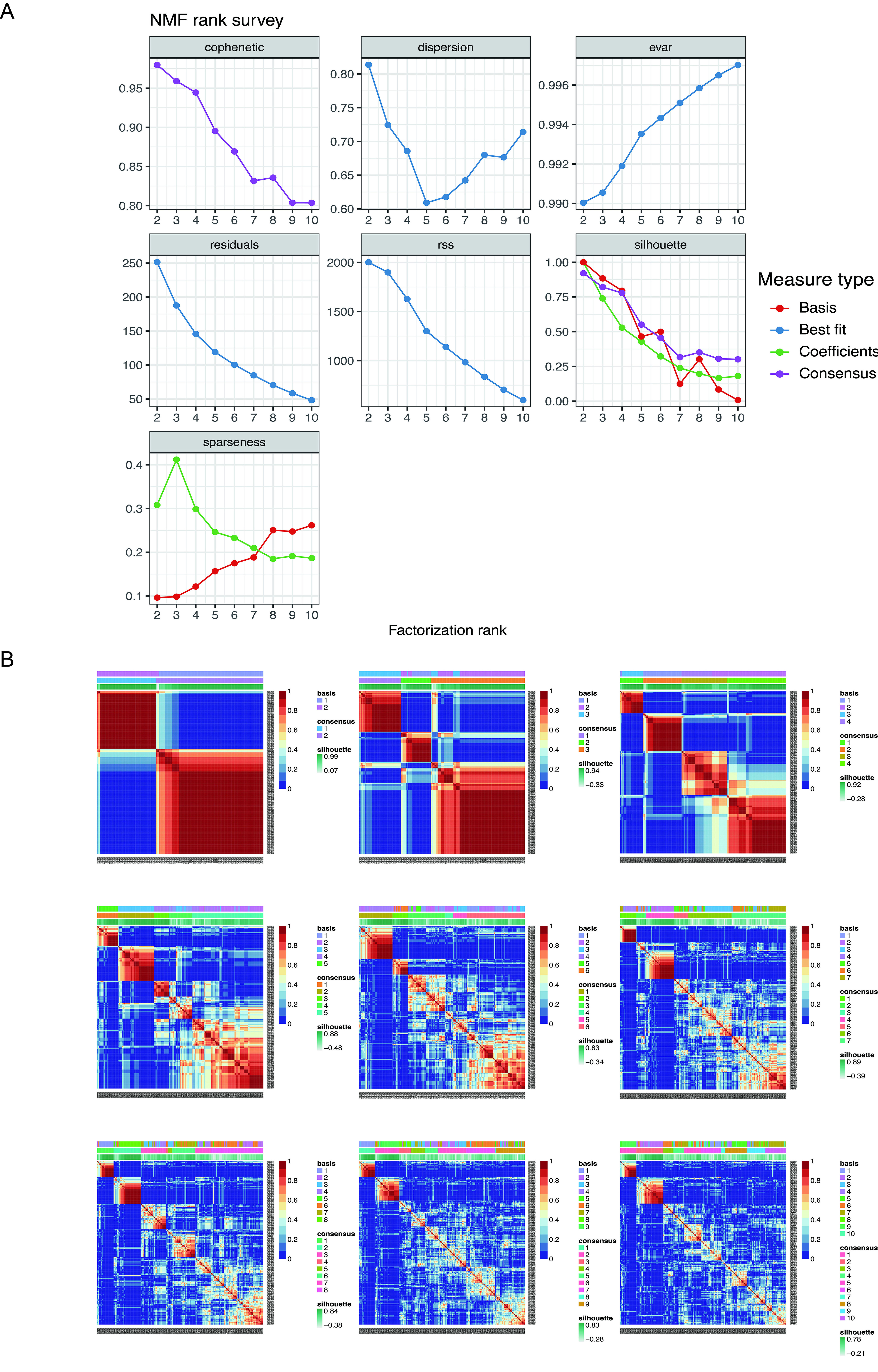

Supplement: Supplementary Figure 1 — NMF clustering was performed on differentially expressed GRGs. (A) Rank survey for nonnegative matrix factorization. (B) Heatmaps of consensus matrices for k=2-9. [file Image1.jpeg]

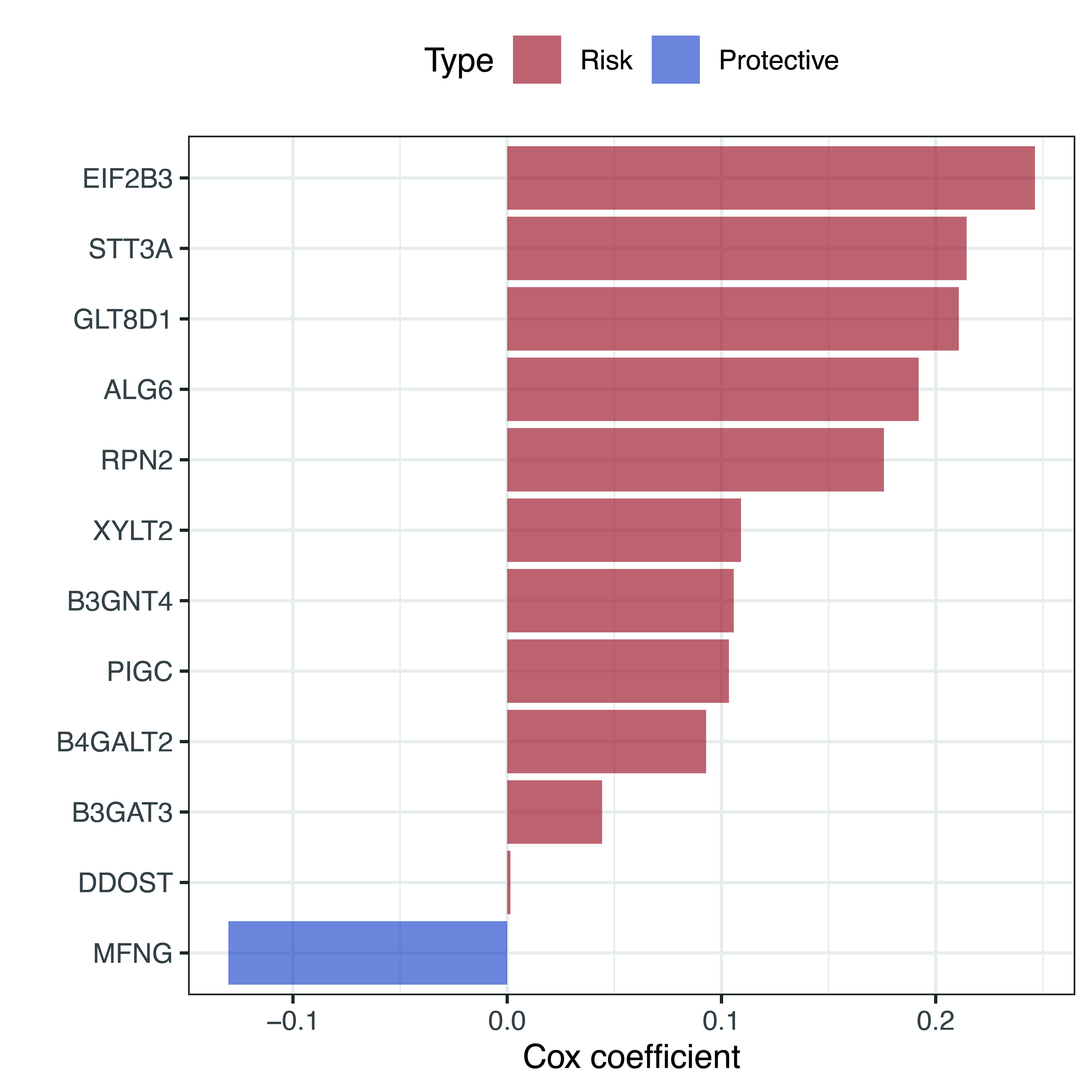

Supplement: Supplementary Figure 2 — Distribution of regression coefficients in the multivariate Cox. This figure illustrates the Cox regression coefficients of the 12 genes included in the glycosylation-related prognostic signature (GRPS). Red bars indicate risk-associated genes (positive coefficients), while blue bars represent protective genes (negative coefficients). The x-axis denotes the magnitude of regression coefficients, and the y-axis lists gene names. [file Image2.jpeg]

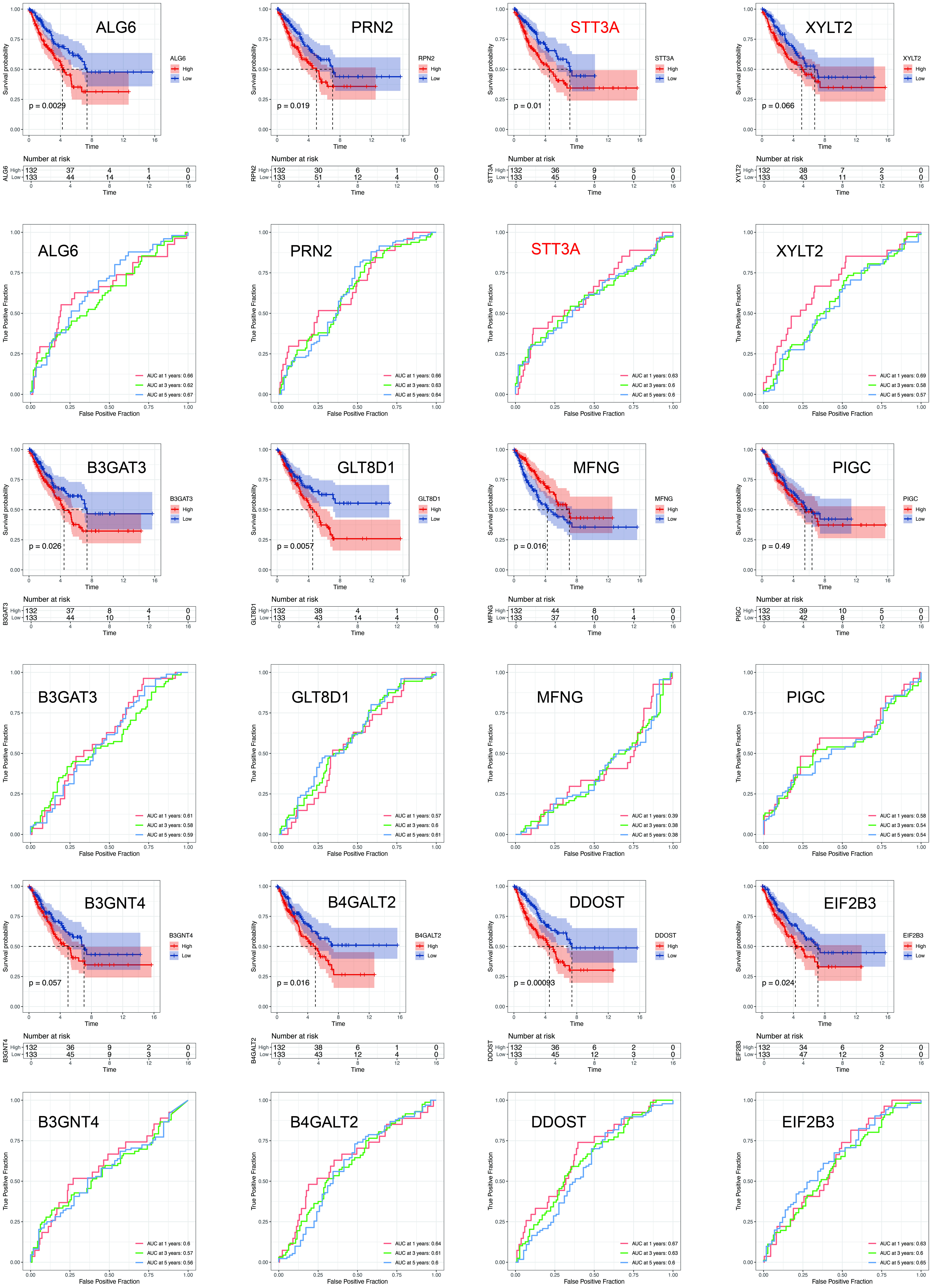

Supplement: Supplementary Figure 3 — Kaplan-Meier survival curves and ROC analyses of 12 GRPS model genes. Prognostic and predictive performance of 12 GRGS model genes via Kaplan-Meier survival and ROC curve analyses. Kaplan-Meier survival plots (top panel) show the stratification of patient groups based on high vs. low expression levels of each GRGS gene, with significant survival differences indicated by log-rank p-values. ROC curves (bottom panel) evaluate the classification power of each gene for 1-, 3-, and 5-year overall survival prediction. The AUC values quantify the sensitivity and specificity of individual genes in the GRGS signature. [file Image3.jpeg]
